# The OsEIL1–OsWOX11 transcription factor module controls rice crown root development in response to soil compaction

**DOI:** 10.1093/plcell/koae083

**Published:** 2024-03-15

**Authors:** Yuxiang Li, Juan Wang, Yadi Gao, Bipin K Pandey, Lucas León Peralta Ogorek, Yu Zhao, Ruidang Quan, Zihan Zhao, Lei Jiang, Rongfeng Huang, Hua Qin

**Affiliations:** Biotechnology Research Institute, Chinese Academy of Agricultural Sciences, Beijing 100081, China; Biotechnology Research Institute, Chinese Academy of Agricultural Sciences, Beijing 100081, China; National Key Facility of Crop Gene Resources and Genetic Improvement, Beijing 100081, China; Biotechnology Research Institute, Chinese Academy of Agricultural Sciences, Beijing 100081, China; Plant and Crop Science Department, School of Biosciences, University of Nottingham, Loughborough LE12 5RD, United Kingdom; Plant and Crop Science Department, School of Biosciences, University of Nottingham, Loughborough LE12 5RD, United Kingdom; National Key Laboratory of Crop Genetic Improvement, Hubei Hongshan Laboratory, Huazhong Agricultural University, Wuhan 430070, China; Biotechnology Research Institute, Chinese Academy of Agricultural Sciences, Beijing 100081, China; National Key Facility of Crop Gene Resources and Genetic Improvement, Beijing 100081, China; Biotechnology Research Institute, Chinese Academy of Agricultural Sciences, Beijing 100081, China; Biotechnology Research Institute, Chinese Academy of Agricultural Sciences, Beijing 100081, China; Biotechnology Research Institute, Chinese Academy of Agricultural Sciences, Beijing 100081, China; National Key Facility of Crop Gene Resources and Genetic Improvement, Beijing 100081, China; Biotechnology Research Institute, Chinese Academy of Agricultural Sciences, Beijing 100081, China; National Key Facility of Crop Gene Resources and Genetic Improvement, Beijing 100081, China

## Abstract

Optimizing the root architecture of crops is an effective strategy for improving crop yields. Soil compaction is a serious global problem that limits crop productivity by restricting root growth, but the underlying molecular mechanisms are largely unclear. Here, we show that ethylene stimulates rice (*Oryza sativa*) crown root development in response to soil compaction. First, we demonstrate that compacted soil promotes ethylene production and the accumulation of ETHYLENE INSENSITIVE 3-LIKE 1 (OsEIL1) in rice roots, stimulating crown root primordia initiation and development, thereby increasing crown root number in lower stem nodes. Through transcriptome profiling and molecular analyses, we reveal that OsEIL1 directly activates the expression of *WUSCHEL-RELATED HOMEOBOX 11* (*OsWOX11*), an activator of crown root emergence and growth, and that *OsWOX11* mutations delay crown root development, thus impairing the plant's response to ethylene and soil compaction. Genetic analysis demonstrates that *OsWOX11* functions downstream of *OsEIL1*. In summary, our results demonstrate that the OsEIL1–OsWOX11 module regulates ethylene action during crown root development in response to soil compaction, providing a strategy for the genetic modification of crop root architecture and grain agronomic traits.

## Teaser

The phytohormone ethylene fine-tunes rice crown root development by activating *WUSCHEL-RELATED HOMEOBOX 11* expression in response to soil compaction.

## Introduction

Roots serve as the interface between the plant and the dynamic soil environment and have crucial functions affecting plant productivity or tolerance to environmental stresses (Lynch 1995; Uga et al. 2013; Kitomi et al. 2020). The root systems of dicots contain a primary root and lateral roots; the continuous growth of the primary root is required for plants to complete their lifecycles (Tian et al. 2014; Zheng et al. 2016). Unlike the primary roots of dicots, the embryonic primary root of monocotyledonous crops, such as rice (*Oryza sativa*), ceases to grow after 7 to 10 d of rapid growth, followed by the gradual emergence and growth of the crown roots (Marcon et al. 2013; Rogers and Benfey 2015; Qin et al. 2022a, 2022b). The crown roots of rice are adventitious roots that initiate from stem nodes or coleoptile sections, forming a major functional component of the mature root system (Zhao et al. 2009; Shao et al. 2019; Kitomi et al. 2020). Therefore, elucidating the molecular mechanisms of crown root development is an important step toward improving root system architecture to enhance yields and other agronomic traits.

Over the years, several critical genes involved in the regulation of crown root development have been identified (Inukai et al. 2005; Zhao et al. 2015; Shao et al. 2019; Zhu et al. 2019). *ADVENTITIOUS ROOTLESS 1/CROWN ROOTLESS 1* (*ARL1/CRL1*), which encodes a LATERAL ORGAN BOUNDARIES (LOB)-domain transcription factor, positively regulates crown root formation and acts as a downstream target of AUXIN RESPONSE FACTOR 1 (ARF1) (Inukai et al. 2005). OsWOX11, a member of the *WUSCHEL-RELATED HOMEOBOX* (*WOX*) gene family, has been reported to interact with ETHYLENE-RESPONSIVE FACTOR 3 (ERF3) to repress the expression of type-A cytokinin response regulator (*RR*) *OsRR2* to control crown root development (Zhao et al. 2009; Zhao et al. 2015). OsWOX11 recruits the ALTERATION/DEFICIENCY IN ACTIVATION 2 (ADA2)–GENERAL CONTROL NON-REPRESSED PROTEIN 5 (GCN5) histone acetyltransferase module to establish cell proliferation programs in the crown root meristem (Zhou et al. 2017). Recent studies have shown that OsWOX11 and CRL1 act synergistically to activate *CYTOKININ OXIDASE 4* (*OsCKX4*) expression to maintain cytokinin homeostasis during crown root development (Geng et al. 2022), suggesting that OsWOX11 might be a key node in the regulation of crown root development.

As the below-ground organ of the plant, root development is greatly influenced by bio-physico-chemical properties of the soil (Bengough et al. 2011; Shekhar et al. 2019; Pandey et al. 2021). Soil compaction is a serious global problem as it causes inadequate rooting and substantial reductions in crop yield (Hamza and Anderson 2005; Correa et al. 2019; Schneider et al. 2021; Pandey and Bennett 2023). Soil compaction restricts root growth by imposing mechanical resistance and reducing soil aeration (Chan et al. 2006). The tillage layer in the paddy field is approximately 15 to 20 cm thick (Zhang et al. 2013), meaning that roots will encounter compacted layers of the soil as they grow deeper. Accumulating investigations show that soil impedance increases ethylene biosynthesis and compacted soil restricts diffusion of ethylene (Zhong et al. 2014; Okamoto and Takahashi 2019; Pandey et al. 2021).

Ethylene is perceived by ethylene receptors at the endoplasmic reticulum membrane and transduced through CONSTITUTIVE TRIPLE RESPONSE 1 (CTR1), ETHYLENE INSENSITIVE 2 (EIN2), and EIN3/EIN3-LIKE 1 (EIL1) (Johnson and Ecker 1998; Zhao et al. 2021). Since EIN3/EIL1 functions as a master transcriptional regulator of the ethylene-signaling pathway (Gagne et al. 2004; An et al. 2010; Salehin and Estelle 2015; Zhao et al. 2021), EIN3/EIL1-dependent transcriptional regulation constitutes a major node of the ethylene response. In rice, ethylene promotes the emergence and growth of adventitious roots from aerial nodes (Lin and Sauter 2018, 2019), implying that ethylene acts as a pivotal internal signal that transduces environmental stimuli in root development; however, the underlying molecular mechanism of ethylene-stimulated root growth is largely unclear.

Here, we report that ethylene functions as a key signal to stimulate crown root development in response to soil compaction. Our findings uncover a link of the OsEIL1–OsWOX11 module in crown root development, making an important contribution to the genetic improvement of crop root systems and the adaptation to compacted soil.

## Results

### Compacted soil stimulates ethylene production and OsEIL1 accumulation to modulate crown root development

Compacted soil layers constrain crop productivity by restricting root growth and exploration in deeper soil profiles (Correa et al. 2019). To investigate the effect of soil compaction on crown root development, we used different concentrations of agar to mimic soils with different levels of compaction. Our results showed that the crown root number increased with increasing agar concentrations (Supplementary Fig. S1A and S1B). Cross-sections of the stem bases showed that fewer crown root primordia were produced by the plants grown on lower agar concentrations compared with the plants grown on higher concentrations of agar (Supplementary Fig. S1C). These results indicate that increasing agar strength stimulates the initiation of crown root primordia in lower stem nodes, thus leading to an increase in crown root number.

Soil impedance stimulates ethylene biosynthesis and soil compaction restricts diffusion of ethylene (Zhong et al. 2014; Okamoto and Takahashi 2019; Pandey et al. 2021). To investigate whether ethylene is involved in regulating crown root development in response to soil compaction, we detected the expression levels of ethylene-responsive genes (ERGs) in roots grown on different concentrations of agar and found that the expression levels of ERGs increased with increasing agar concentrations (Supplementary Fig. S1D). Correspondingly, ethylene production also increased with increasing agar concentrations (Supplementary Fig. S1E). Consistent with the previous findings that ethylene promotes the accumulation of EIN3/EIL1 in *Arabidopsis thaliana* (Gagne et al. 2004; An et al. 2010), ethylene treatment substantially resulted in the accumulation of OsEIL1 in rice roots (Supplementary Fig. S1F and S1G). We also examined OsEIL1 protein levels in roots grown on different concentrations of agar. As expected, OsEIL1 accumulation increased with increasing agar concentrations (Supplementary Fig. S1H). These results indicate that increasing soil compaction leads to ethylene accumulation in roots, thereby affecting crown root development.

To assess the role of ethylene on crown root development, we observed crown root development in ethylene-signaling mutants using 10-d-old seedlings grown in the absence or presence of ethylene. In the absence of ethylene, the *ein2* and *eil1* mutants exhibited fewer crown roots than the wild-type Nipponbare (Nip), whereas constitutive overexpressing *OsEIN2/OsEIL1* (*EIN2-OX* and *EIL1-OX*, respectively) resulted in increased crown root number compared with Nip (Supplementary Fig. S2A to S2D). Ethylene treatment significantly increased the crown root number in Nip plants, but this effect was attenuated in *ein2* and *eil1* mutants (Supplementary Fig. S2A and S2C). Cross-sections of the stem bases showed that the formation of crown root primordia was substantially retarded in *ein2* and *eil1* plants compared with Nip (Supplementary Fig. S2E). By contrast, the crown root primordia of *EIN2-OX* and *EIL1-OX* plants appeared much earlier and grew more rapidly compared with Nip (Supplementary Fig. S2F). Ethylene treatment had a substantial promotive effect on the development of crown root primordia in Nip. However, this effect was attenuated in the *ein2* and *eil1* mutants (Supplementary Fig. S2E). These results indicate that ethylene stimulates the initiation and development of crown root primordia in lower stem nodes.

**Figure 1.**
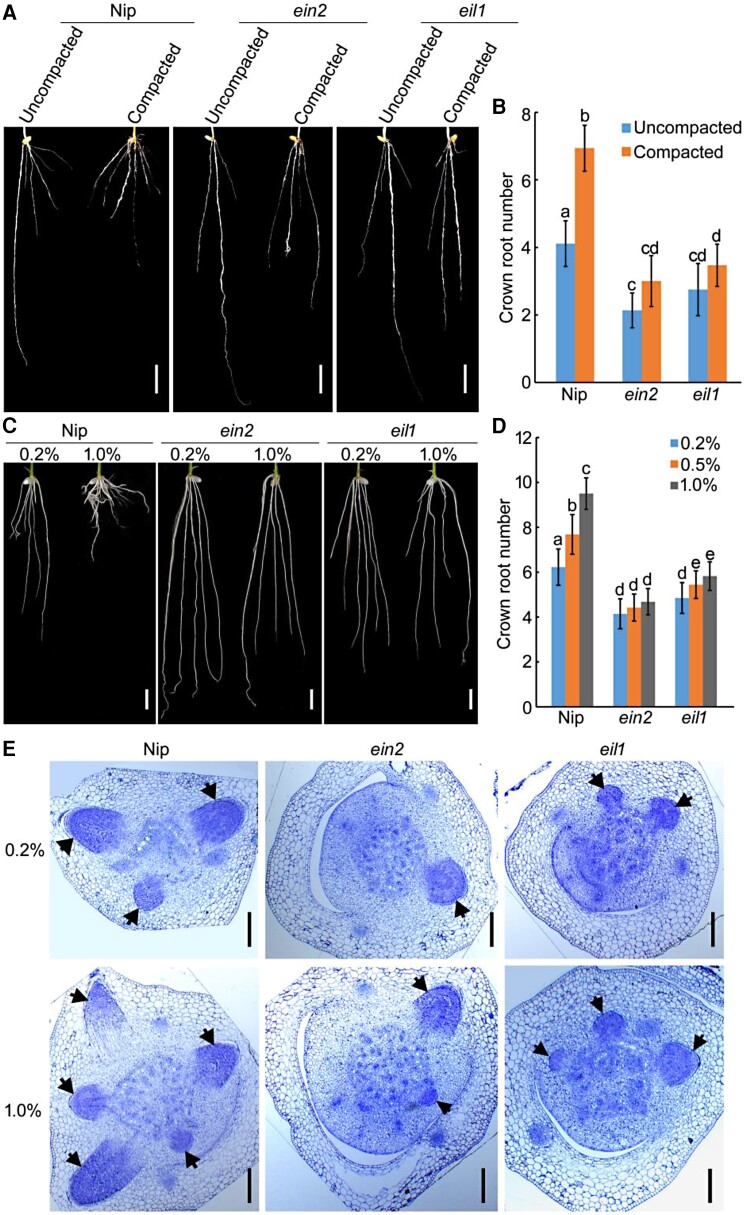
Ethylene-signaling pathway is required for soil compaction-modulated crown root development. **A)** and **C)** Root phenotypes of 10-d-old Nipponbare (Nip), *ein2*, and *eil1* seedlings grown in uncompacted and compacted soil A) or on different concentrations of agar C). Bar = 10 mm. **B)** and **D)** Crown root number of plants shown in A) and C). The values are means ± SD of 20 to 30 independent seedlings per sample. Different letters indicate significant differences (*P* < 0.05, one-way ANOVA with Tukey's test). **E)** Representative toluidine blue-stained cross sections of the stem base of 4-d-old Nip, *ein2*, and *eil1* seedlings grown on different agar concentrations. Arrows indicate crown root primordium. Bars = 100 *μ*m.

Next, we examined the crown roots of *ein2* and *eil1* mutants using 10-d-old seedlings grown in uncompacted or compacted soil conditions. Our observations showed that compacted soil significantly increased the crown root number in Nip plants, but this effect was weakened in *ein2* and *eil1* mutants (Fig. 1A and 1B). Moreover, compacted soil significantly reduced the shoot length and fresh weight of shoots in Nip seedlings, with a milder phenotype in *ein2* and *eil1* mutants (Supplementary Fig. S3), indicating that soil compaction retards seedling growth by modulating root development, and the ethylene-signaling pathway is crucial for this process.

Similar to those of soil compaction, increasing agar concentration also increased the crown root number in Nip, however, this regulatory effect was attenuated in *ein2* and *eil1* mutants (Fig. 1C and 1D). Furthermore, anatomical analysis of stem bases revealed that an increase in agar concentration stimulated the initiation and development of crown root primordia in Nip, but this tendency was weakened in *ein2* and *eil1* mutants (Fig. 1E). These results further reveal that ethylene is essential for the development of crown root in response to soil compaction, and soil compaction stimulates crown root development depending on ethylene-signaling pathway.

Furthermore, field-grown *ein2* and *eil1* plants had fewer crown roots, whereas *EIN2-OX* and *EIL1-OX* plants had more crown roots and shorter shoots, compared with the wild type (Supplementary Fig. S4A and S4B). Importantly, grain length and grain width in mature plants were increased in *EIN2-OX* and *EIL1-OX* plants, however, in *ein2* and *eil1* mutants there was no significant impact on grain length and width, compared with that of Nip (Supplementary Fig. S4C to S4F). While the thousand-grain weight significantly increased in *EIN2-OX* and *EIL1-OX* plants, whereas no obvious changes were observed in the *ein2* and *eil1* mutants (Supplementary Fig. S4G). Notably, the grain yield per plant was reduced in *ein2* mutants and *EIN2-OX* and *EIL1-OX* plants (Supplementary Fig. S4H), perhaps due to the negative effect of ethylene on grain filling (Ma et al. 2013; Sekhar et al. 2015). These results indicate that ethylene is an important regulator of root development in rice, but knocking out or enhancing the expression of ethylene-related regulators impacts root development and grain yield, implying that exploring favorable alleles of ethylene-related genes might balance both root development and yield.

### Transcriptome profiling reveals that OsWOX11 might be involved in ethylene-stimulated crown root development

**Figure 2.**
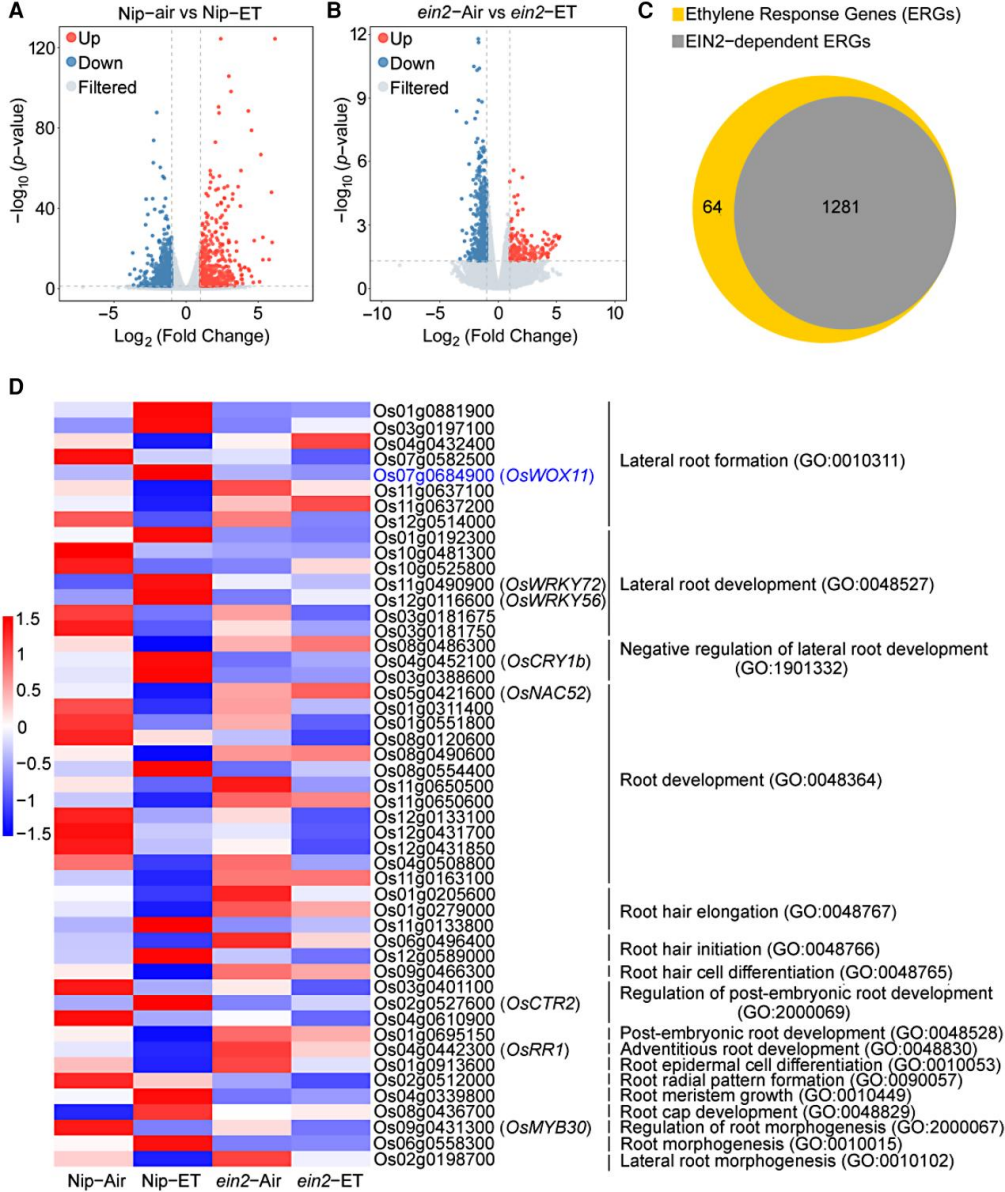
Transcriptome analysis of differentially expression genes (DEGs) regulated by ethylene in Nipponbare (Nip) and *ein2* roots. **A)** and **B)** Volcano plots show DEGs in Nip A) and *ein2* B) roots with or without 10 *μ*L/L ethylene (ET) treatment. The blue and red dots represent downregulated DEGs with log_2_ (FC) < −1 and upregulated DEGs with log_2_ (FC) > 1, respectively. The gray dots represent no significant difference in expression. **C)** Venn diagram showing OsEIN2-dependent ethylene-response genes (ERGs). **D)** Heat map of microarray expression profiles for genes associated with root development.

To understand the molecular mechanisms underlying ethylene-stimulated crown root development, we performed transcriptome analysis using mRNAs prepared from the roots of *ein2* and Nip plants with or without ethylene treatment. OsEIN2 is a central component of ethylene signaling, and mutation in *OsEIN2* leads to ethylene insensitivity in both root and coleoptile growth (Ma et al. 2013). The threshold for significantly differentially expressed genes (DEGs) was set at a log_2_-scale fold change (FC) value of more than 1 or less than ‒1 and adjusted to *P* < 0.05. Using these criteria, we identified 1,345 DEGs in Nip and 783 DEGs in *ein2* (Supplementary Data Set 1). These DEGs altered by ethylene are shown in the volcano plots, which illustrate the asymmetry between upregulated (red) and downregulated (blue) DEGs (Fig. 2A and 2B). Venn diagram analysis showed that 95% (1,281) ethylene-regulated genes (ERGs) in Nip were OsEIN2-dependent ERGs (Fig. 2C, Supplementary Data Set 2), which is consistent with the fact that OsEIN2 is a central component of ethylene signaling in rice. Gene ontology (GO) enrichment analysis showed that these OsEIN2-dependent ERGs were related to dioxygenase activity, UDP-glucosyltransferase activity, gibberellin biosynthetic process, defense response, and ethylene-activated signaling pathway (Supplementary Fig. S5), suggesting that ethylene is involved in a diverse range of molecular functions, cellular components, and biological processes.

We further analyzed genes associated with root development in these OsEIN2-dependent ERGs by tree view (heat map) (Fig. 2D). Among 49 genes subjected to heat map analysis, the functions of most genes have not been reported, only *OsCTR2*, *OsRR1*, and *OsWOX11* were reported to regulate crown root development (Zhao et al. 2009; Kitomi et al. 2011; Wang et al. 2013). OsCTR2 is a negative regulator of ethylene signaling and acts upstream of OsEIN2 (Wang et al. 2013); OsRR1 is a negative regulator of cytokinin signaling and its expression is repressed in *OsWOX11*-overexpressing plants (Zhao et al. 2009; Kitomi et al. 2011); OsWOX11 has been reported to control crown root development by directly regulating cytokinin signaling and homeostasis (Zhao et al. 2009; Zhao et al. 2015; Geng et al. 2022). Based on these studies, we chose OsWOX11 for further investigations. Heat map analysis showed that *OsWOX11*, associated with lateral root formation (GO: 0010311) category, was upregulated by ethylene treatment in Nip but not in *ein2* (Fig. 2D). These results indicate that ethylene may stimulate crown root development by activating *OsWOX11* transcription.

### OsWOX11 is required for ethylene- and soil compaction-stimulated crown root development

To explore the potential involvement of OsWOX11 in ethylene-promoted crown root development, we generated loss-of-function mutants of *OsWOX11* via CRISPR–Cas9 (*oswox11*), as confirmed by sequencing the target gene. The *oswox11-1* and *oswox11-3* mutants contained 4-bp and 7-bp deletions in the coding regions of the target genes, respectively, resulting in early termination, whereas the *oswox11-2* mutant contained 1-bp insertion in the coding region, triggering a frameshift in the open reading frame (Supplementary Fig. S6A and S6B). We also generated overexpression (OX) lines containing *OsWOX11* coding sequence under the control of the *CaMV35S* promoter; the increased expression of *OsWOX11* was confirmed by reverse transcription quantitative PCR (RT-qPCR, Supplementary Fig. S6C). Although the expression of *OsWOX11* was significantly increased in *OsWOX11-OX* plants, OsWOX11 protein was increased slightly in *OsWOX11-OX* lines (Supplementary Fig. S6D), implying that OsWOX11 is a key regulator in plant development and plants must maintain proper levels of OsWOX11 protein to ensure normal growth.

Overexpressing *OsWOX11* resulted in an increase in crown root number and grain length; whereas the *oswox11* mutants had fewer crown roots and slightly reduced grain length and grain width compared with the wild type. The plant height was significantly reduced in the *OsWOX11-OX* plants and loss-of-function mutants compared with the wild type, whereas grain width was not affected in the *OsWOX11-OX* plants (Supplementary Figs. S6E to S6H and S7A to S7F). Further analyses with measurements of the thousand-grain weight showed that *OsWOX11-OX* plants had slight increases, whereas the *oswox11* mutants showed significant decreases in this parameter (Supplementary Fig. S7G). Moreover, the grain yield per plant was reduced in *oswox11* mutants and *OsWOX11-OX* plants (Supplementary Fig. S7H). These results indicate that disruption of *OsWOX11* expression affects crown root development and yield-related traits in rice.

Next, we treated seedlings of the *OsWOX11-OX* lines and *oswox11* mutants with ethylene. Ethylene treatment increased crown root number in the wild type and the *OsWOX11-OX* lines (Supplementary Fig. S8A and S8B). Ethylene has a similar effect on the number of crown roots in wild type and *OsWOX11-OX* lines, suggesting that stimulation of ethylene in crown root development is not simply due to an increase in the transcription of *OsWOX11*, and other regulations or other downstream genes were also involved in ethylene-stimulated crown root development. In contrast, the crown root number of the *oswox11* mutants remained unchanged by ethylene treatment as compared with the untreated control (Supplementary Fig. S8C and S8D), revealing that a functional OsWOX11 is required for ethylene-stimulated crown root development.

**Figure 3.**
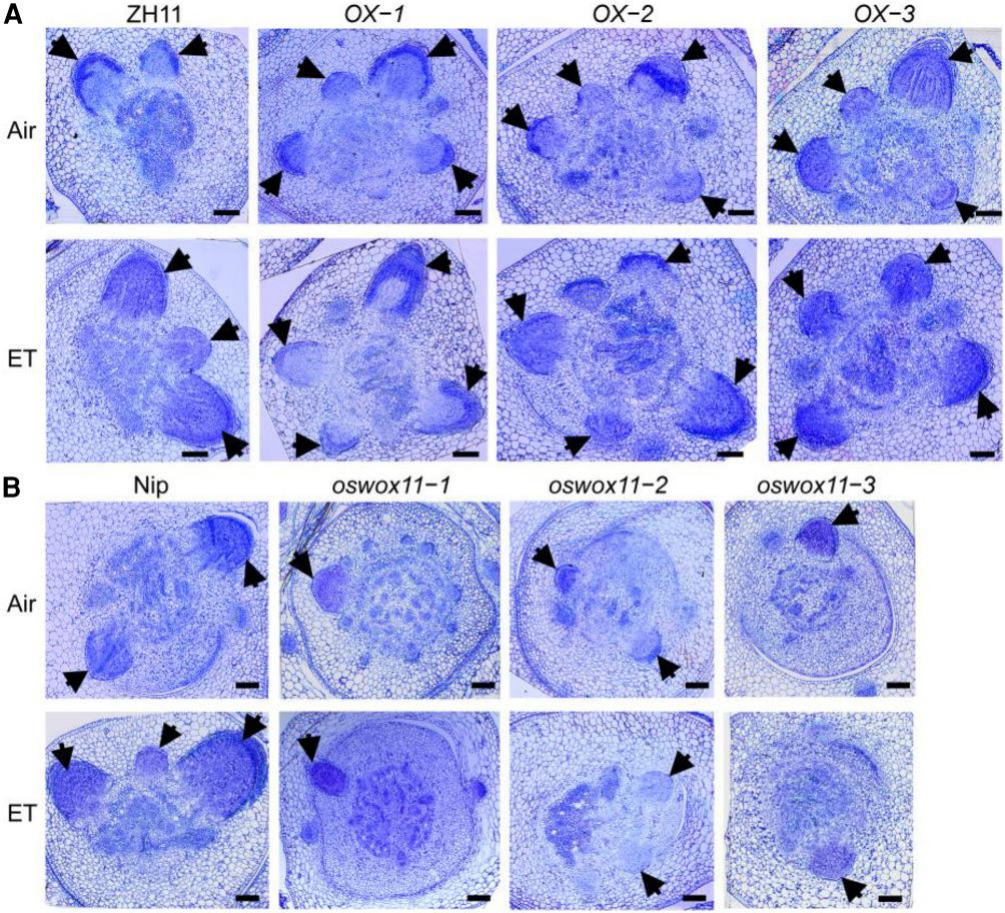
*OsWOX11*-mediated pathway is required for ethylene-induced crown root primordium initiation and development. **A)** and **B)** Representative toluidine blue-stained cross sections of the stem base of 4-d-old *OsWOX11* overexpression (OX) plants and *oswox11* mutants with or without 10 *μ*L/L ethylene (ET) treatment. Arrows indicate crown root primordium. Bars = 100 *μ*m. Nip represents Nipponbare and ZH11 represents Zhonghua 11.

Under normal conditions, the crown root primordia of *OsWOX11-OX* plants grew much earlier and more rapidly than those of the wild type, whereas the initiation of crown root primordia was retarded in *oswox11* plants (Fig. 3A and 3B). Ethylene treatment substantially induced the initiation and development of crown root primordia in *OsWOX11-OX* plants, whereas the crown root primordia in *oswox11* plants were not responsive to ethylene treatment (Fig. 3A and 3B). Further examining the ethylene response in T-DNA insertion line of *OsWOX11* (*oswox11-4*) also confirmed that ethylene-stimulated crown root development was impaired in *oswox11-4* plants (Supplementary Fig. S9). These results suggest that OsWOX11 plays an important role in crown root development and that the effect of ethylene on crown root development occurs through multiple pathways, including the *OsWOX11*-mediated pathway.

**Figure 4.**
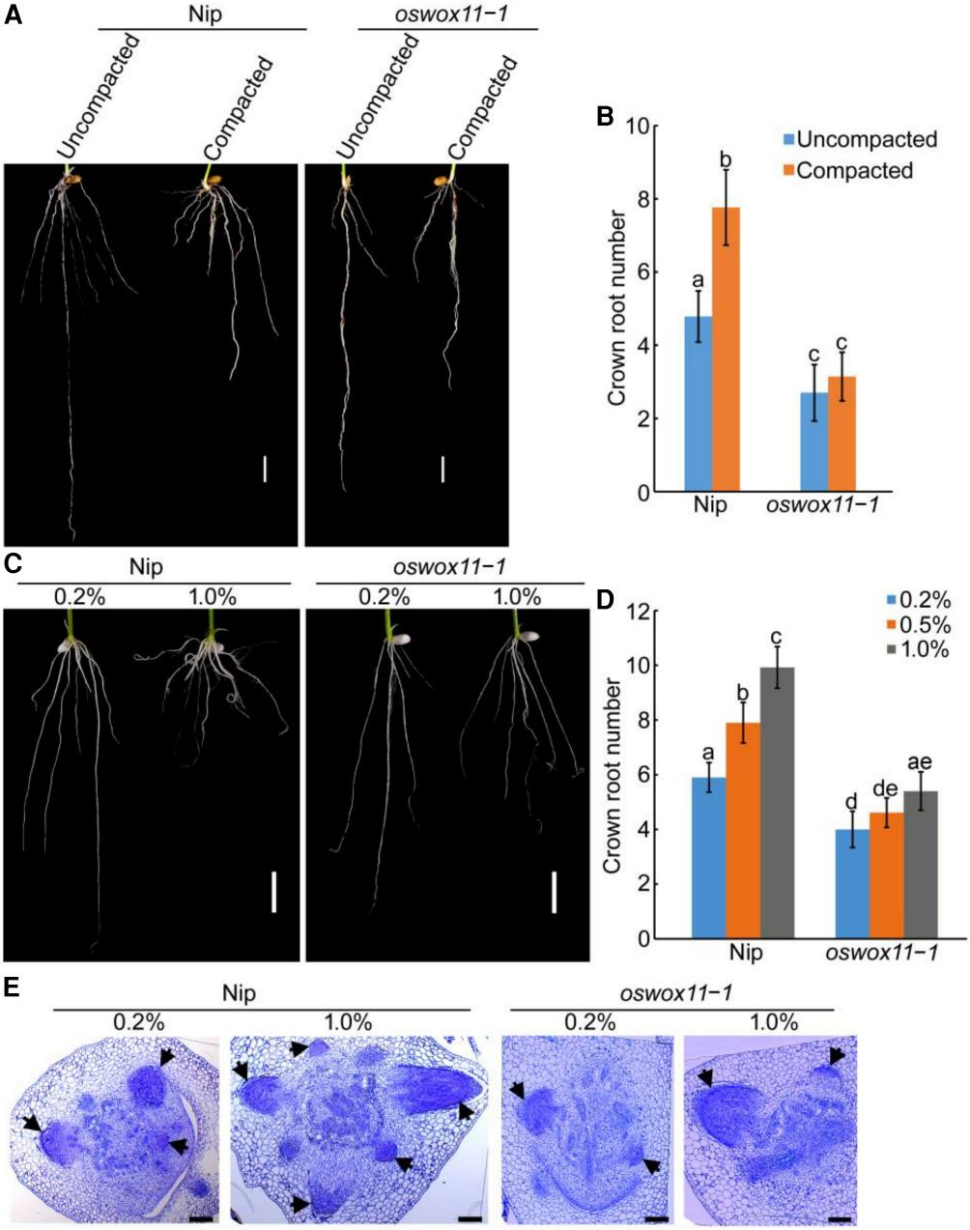
*OsWOX11*-mediated pathway is required for soil compaction-modulated crown root development. **A)** and **C)** Root phenotypes of 10-d-old Nipponbare (Nip) and *oswox11-1* seedlings grown in uncompacted and compacted soil A) or on different concentrations of agar C). Bar = 10 mm. **B)** and **D)** Crown root number of plants shown in A) and C). The values are means ± SD of 20 to 30 independent seedlings per sample. Different letters indicate significant differences (*P* < 0.05, one-way ANOVA with Tukey's test). **E)** Representative toluidine blue-stained cross sections of the stem base of 4-d-old Nip and *oswox11-1* seedlings grown on different agar concentrations. Arrows indicate crown root primordium. Bars = 100 *μ*m.

Subsequently, we analyzed the crown root development of *oswox11* in response to soil compaction. Our results showed that soil compaction-stimulated crown root development was abolished in *oswox11-1* plants (Fig. 4A and 4B). Moreover, compacted soil significantly reduced the shoot length and fresh weight of shoots in Nip seedlings, with a milder phenotype in *oswox11-1* plants (Supplementary Fig. S10). Similar to the effects observed in soil compaction, the number of crown roots and promotion of crown root primordia development increased in Nip with rising agar concentration, but this phenotype was weakened in *oswox11-1* mutant (Figs. 4C to 4E). These results indicate that an *OsWOX11*-mediated pathway is required for soil compaction-stimulated crown root and shoot development.

### OsEIL1 directly binds to the promoter regions of *OsWOX11* to activate its expression

**Figure 5.**
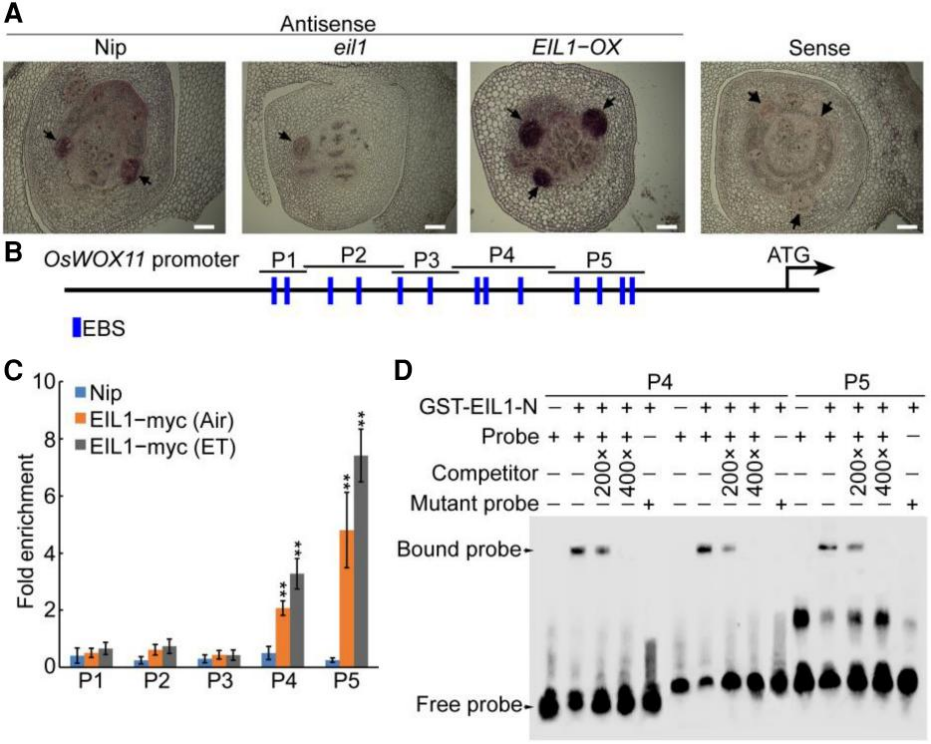
OsEIL1 directly binds to *OsWOX11* promoter region to activate its expression. **A)** In situ hybridization detection of *OsWOX11* transcripts in the crown root primordia in the Nipponbare (Nip), *eil1*, and *EIL1-OX* (overexpressing *OsEIL1*) seedlings with an antisense or sense probe (control). Arrows indicate crown root primordium. Bars = 100 *μ*m. **B)** Schematic diagram of putative OsEIL1-binding site (EBS) in the promoter of *OsWOX11*. Blue boxes indicate the position of the EBS. P1–P5 are *OsWOX11* promoter fragments. **C)** Anti-myc ChIP assays with DNA from 10-d-old seedling roots of Nip and overexpressing *OsEIL1* with myc-tag (*EIL1-myc*) transgenic plants. ET represents ethylene treatment. ** indicate significant differences by Student's *t*-test compared to Nip at *P* < 0.01. **D)** EMSA using normal (ATGTA/TACAT) and mutated EBS (GGAGC) in P4 and P5 with glutathione-S-transferase-tagged OsEIL1 N-terminal fusion protein (GST-EIL1-N). GST-tag was used in place of GST-EIL1-N for no-protein controls. Competition was done by adding an excess of unlabeled probe (Competitor). Three biological replicates were performed with similar results.

*OsWOX11* was identified as an OsEIN2-dependent ERG, prompting us to determine whether OsEIL1 regulates transcript levels of *OsWOX11* as well. To address this, first we performed RT-qPCR to measure the expression levels of *OsWOX11* in the *ein2* and *eil1* mutants following ethylene treatment. Exogenous ethylene treatment increased *OsWOX11* transcript levels in Nip plants. However, this effect was greatly reduced in *ein2* and *eil1* plants (Supplementary Fig. S11A). In the absence of ethylene, the expression of *OsWOX11* was significantly lower in *ein2* and *eil1* plants but higher in *EIN2-OX* and *EIL1-OX* plants as compared with Nip (Supplementary Fig. S11B). Next, these results were further confirmed by in situ hybridization assay (Fig. 5A). These results demonstrate that ethylene transcriptionally activates the expression of *OsWOX11* primarily mediating the ethylene-signaling pathway.

Previous studies have shown that OsWOX11 directly binds to *OsRR2* and *OsCKX4* to regulate their expression during crown root development (Zhao et al. 2009; Geng et al. 2022). Hence, we also determined *OsRR2* and *OsCKX4* expression in the *ein2* and *eil1* mutants in response to ethylene treatment. Our results showed that ethylene treatment suppressed *OsRR2* expression and induced *OsCKX4* expression in Nip plants, and this effect was weakened in *ein2* and *eil1* plants (Supplementary Fig. S12A and S12C). In addition, the expression of *OsRR2* was significantly higher in *ein2* and *eil1* plants but lower in *EIN2-OX* and *EIL1-OX* plants compared with Nip (Supplementary Fig. S12B). In contrast, the expression of *OsCKX4* was significantly decreased in *ein2* and *eil1* plants but increased in *EIN2-OX* and *EIL1-OX* plants compared with Nip (Supplementary Fig. S12D).

We further checked the expression of *OsRR2* and *OsCKX4* in the *oswox11* mutant with or without ethylene treatment. Our results showed that ethylene treatment suppressed *OsRR2* expression and induced *OsCKX4* expression in Nip plants, and this effect was weakened in *oswox11* mutant (Supplementary Fig. S13). These results suggest that ethylene-mediated upregulation of *OsWOX11* is responsible for the increase in the expression of *OsCKX4* and the reduction in the levels of *OsRR2*, and *OsWOX11*-mediated pathway is involved in ethylene-regulated crown root development.

To determine whether OsEIL1 functions as a direct regulator of *OsWOX11*, we analyzed the promoter sequences of *OsWOX11* and identified 13 putative OsEIL1-binding sites (EBS: ATGTA/TACAT) (Yang et al. 2015) in the *OsWOX11* promoter (Fig. 5B), implying that *OsWOX11* might be a downstream target of OsEIL1. Hence, we performed a chromatin immunoprecipitation (ChIP) assay using transgenic plants harboring myc-tagged OsEIL1 (OsEIL1-myc). As shown in Fig. 5C, OsEIL1 was significantly enriched in the P4 and P5 fragments of the *OsWOX11* promoter, and ethylene treatment enhanced OsEIL1 binding to the promoter regions of *OsWOX11*, while there was no significant enrichment in the other fragments (Fig. 5C).

Subsequently, we conducted an electrophoretic mobility shift assay (EMSA) using the GST-EIL1-N fusion protein expressed in *Escherichia coli*. The results, as shown in Fig. 5D, demonstrated that the GST-EIL1-N fusion protein was specifically bound to DNA probes containing the EBS motif. This motif was present in the P4 and P5 fragments of the *OsWOX11* promoter. However, the fusion protein did not bind to the probes with mutated EBS motifs. The specificity of this binding was confirmed by a competition assay using unlabeled competitor probe (Fig. 5D). These results indicate that OsEIL1 directly binds to the *OsWOX11* promoter in vitro and in vivo.

To determine whether OsEIL1 activates the expression of *OsWOX11*, we performed a transient expression assay in which we fused the 2000-bp promoter sequence upstream of the ATG codon of *OsWOX11* to the *LUCIFERASE* (*LUC*) reporter gene and co-transfected *Nicotiana benthamiana* leaves and rice protoplasts with the effector plasmid containing *Pro35S:EIL1*. The presence of the effector significantly increased LUC activity driven by the *OsWOX11* promoter compared with the control vector (Supplementary Fig. S14). These results indicate that OsEIL1 activates the expression of *OsWOX11*.

### The OsEIL1–OsWOX11 module is required for ethylene- and soil compaction-regulated crown root development

**Figure 6.**
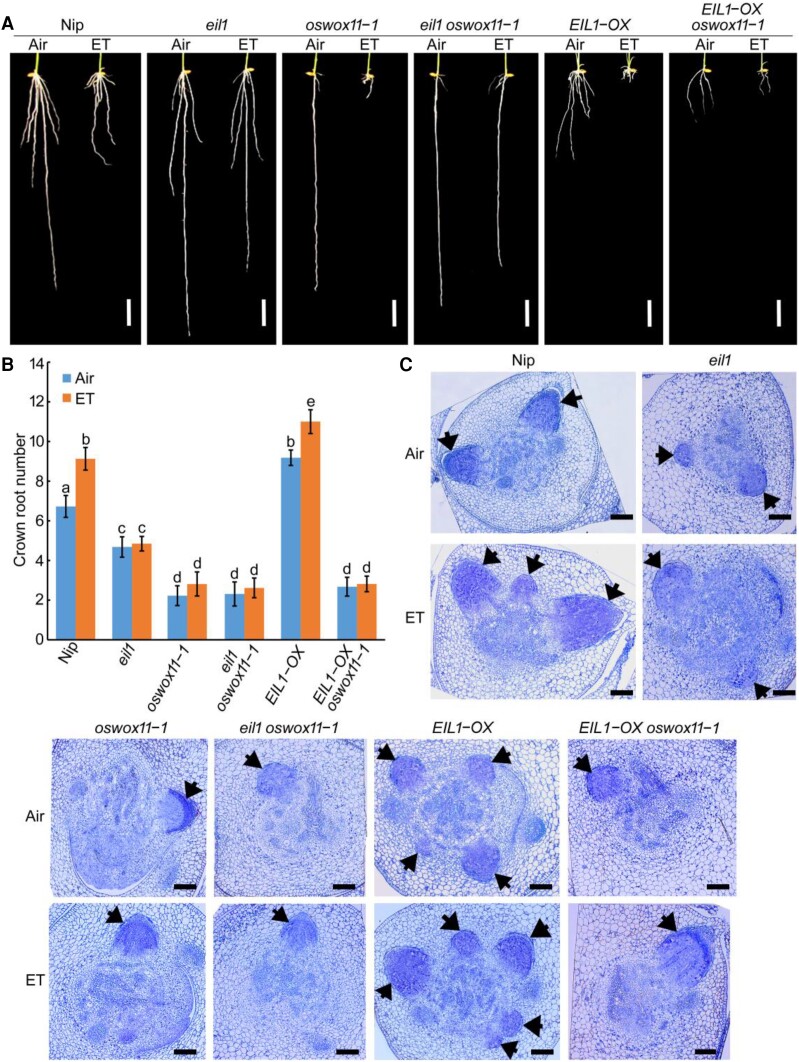
OsWOX11 genetically functions downstream of *OsEIL1* to regulate crown root development. **A)** Root phenotypes of 10-d-old Nipponbare (Nip), *eil1*, *oswox11-1*, *eil1 oswox11-1*, *EIL1-OX* (overexpressing *OsEIL1*), and *oswox11-1 EIL1-OX* seedlings with or without 10 *μ*L/L ethylene (ET) treatment. Bar = 10 mm. **B)** Crown root number of plants shown in A). Each column is an average of 20 to 30 independent seedlings and bars indicate ± SD. Different letters indicate significant differences (*P* < 0.05, one-way ANOVA with Tukey's test). **C)** Representative toluidine blue-stained cross sections of the stem base of 4-d-old Nip, *eil1*, *oswox11-1*, *eil1 oswox11-1*, *EIL1-OX*, and *oswox11-1 EIL1-OX* seedlings with or without 10 *μ*L/L ethylene treatment. Arrows indicate crown root primordium. Bars = 100 *μ*m.

To examine the genetic relationship between *OsWOX11* and the ethylene-signaling component *OsEIL1*, we generated *eil1 oswox11-1* plants by crossing homozygous *oswox11-1* with *eil1*. The crown root number of *eil1 oswox11-1* double mutant was similar to *oswox11-1* mutant and significantly less than Nip and *eil1* plants (Fig. 6A and 6B). Moreover, ethylene-stimulated increase in crown root number was completely abolished in the *eil1 oswox11-1* double mutant (Fig. 6A and 6B). Anatomical analysis of stem bases revealed that crown root primordium development was severely retarded in the *eil1 oswox11-1* double mutant, and ethylene treatment did not promote crown root primordia initiation and development in *eil1 oswox11-1* double mutant (Fig. 6C). These results suggest that *OsWOX11* and *OsEIL1* function in the same pathway for ethylene-stimulated crown root initiation.

To further examine the genetic relationship between *OsWOX11* and *OsEIL1*, we analyzed the ethylene response of the *oswox11-1 EIL1-OX* plants that were obtained by crossing homozygous *oswox11-1* with *EIL1-OX*. The expression of *OsEIL1* in the *oswox11-1 EIL1-OX* plants was examined by RT-qPCR (Supplementary Fig. S15A). The crown root number and crown root primordia of *oswox11-1 EIL1-OX* plants resembled those of *oswox11-1* with or without ethylene treatment (Fig. 6). The grain size was further examined in Nip, *oswox11-1*, *EIL1-OX*, and *oswox11-1 EIL1-OX* plants using well-filled grains. The results showed that the effects on grain size triggered by the overexpression of *OsEIL1* were suppressed in the *oswox11-1* mutant (Supplementary Fig. S15B to S15F). These results indicate that *OsWOX11* acts downstream of the ethylene-signaling pathway and that the *OsWOX11*-mediated pathway is required for the regulation of ethylene-stimulated crown root development and grain size via OsEIL1 signaling.

**Figure 7.**
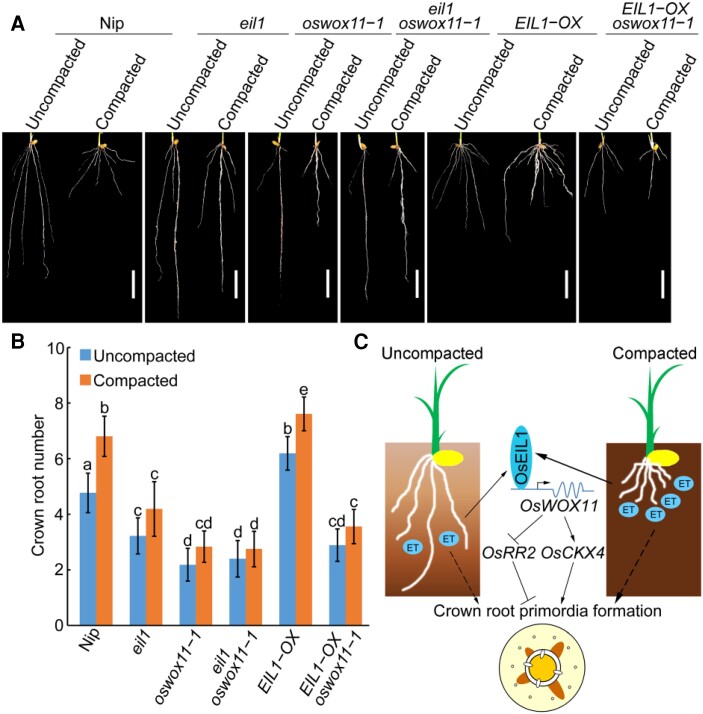
OsEIL1-OsWOX11 module regulates crown root development in response to soil compaction. **A)** Root phenotypes of 10-d-old Nipponbare (Nip), *eil1*, *oswox11-1*, *eil1 oswox11-1*, *EIL1-OX* (overexpressing *OsEIL1*), and *oswox11-1 EIL1-OX* seedlings grown in uncompacted and compacted soil conditions. Bar = 10 mm. **B)** Crown root number of plants shown in A). Each column is an average of 20 to 30 independent seedlings and bars indicate ± SD. Different letters indicate significant differences (*P* < 0.05, one-way ANOVA with Tukey's test). **C)** Schematic representation of root responses in compacted soil *vs* uncompacted soil. Compacted soil stimulates ethylene (ET) biosynthesis and restricts ethylene diffusion, which leads to the accumulation of OsEIL1 levels in roots. OsEIL1 in turn activates the expression of *OsWOX11*, and *OsWOX11* further regulates *OsRR2* and *OsCKX4* to modulate cytokinin signaling and homeostasis to promote crown root primordium initiation and development. In parallel, other unknown pathways are also involved in ethylene-stimulated crown root primordium initiation and development. Ultimately increasing crown root number. The solid lines indicate direct interactions, and the dashed lines indicate indirect interactions. The arrows indicate stimulatory effects, whereas the flat arrows indicate inhibitory effects.

To investigate the OsEIL1-OsWOX11 module in crown root development in response to soil compaction, we detected the expression levels of *OsWOX11* in Nip, *ein2*, and *eil1* plants under uncompacted and compacted soil conditions. Our results showed that soil compaction increased *OsWOX11* transcript levels in Nip plants. However, this effect was weakened in *ein2* and *eil1* mutants (Supplementary Fig. S16), indicating that OsEIL1 activates the expression of *OsWOX11* gene in mediating compacted soil-stimulated crown root development. We further examined the root phenotype of Nip, *eil1*, *oswox11-1*, *eil1 oswox11-1*, *EIL1-OX*, and *oswox11-1 EIL1-OX* plants grown in uncompacted and compacted soil conditions. Soil compaction significantly increased the crown root number in Nip and *EIL1-OX* plants, whereas this effect was weakened in *eil1*, *oswox11-1*, *eil1 oswox11-1*, and *oswox11-1 EIL1-OX* plants (Fig. 7A and 7B). These results indicate that soil compaction stimulates crown root development via the OsEIL1–OsWOX11 module.

## Discussion

Plants face a substantial challenge due to the complexity of the environment in which they must survive. Root systems are vital for addressing this complexity. To ensure an optimal response to changing environmental situations, roots are continuously reshaped by the initiation and elongation of new roots throughout the growth period, and phytohormones act as all-encompassing regulators in this process (Steffens et al. 2006; Pandey et al. 2021; Huang et al. 2022; Qin et al. 2022a). In the present study, we further demonstrate that ethylene is an important regulator of crown root development in response to soil compaction, and OsEIL1 plays an essential role in the initiation and development of crown root primordia by activating *OsWOX11* expression. Thus, our findings reveal a OsEIL1–OsWOX11 module regulating ethylene-mediated crown root development in compacted soil, providing key insights for the development of rice varieties with improved abilities to acquire soil resources efficiently.

Crown roots are the main component of the fibrous root system in cereal crops. Modulation of crown root growth could enhance crop yields and improve the plant's ability to withstand various adversities (Uga et al. 2013; Kitomi et al. 2020). Mounting evidence indicates that ethylene plays a crucial role in promoting the emergence and growth of adventitious roots in rice (Lin and Sauter 2018, 2019), but the underlying molecular mechanism is largely unclear. In this study, we uncovered that ethylene transcriptionally regulates the expression of *OsWOX11*, a key regulator of crown root development (Zhao et al. 2009, 2015; Zhou et al. 2017), thereby promoting the initiation and development of crown root primordia. This conclusion is supported by the following evidence: (i) exogenous ethylene treatment increased crown root number and promotes crown root primordium initiation and development; (ii) ethylene induces *OsWOX11* transcription and overexpressing of *OsWOX11* resulted in increased crown root number; (iii) knockout of *OsWOX11* repressed ethylene-stimulated initiation and development of crown root primordia; (iv) ethylene regulates the expression of *OsWOX11* depending on OsEIL1, and OsEIL1 directly binds to the *OsWOX11* promoter to activate its expression; (v) ethylene-suppressed *OsRR2* expression and ethylene-induced *OsCKX4* expression was weakened in *ein2*, *eil1*, and *oswox11* mutant; and (vi) both OsEIL1 and OsWOX11 positively affect crown root development, and genetic analysis suggests that ethylene signaling acts upstream of the *OsWOX11*-mediated pathway to regulate crown root development. Thus, we propose that the effect of ethylene on crown root development might occur through multiple pathways, including the *OsWOX11*-mediated pathway, where ethylene primarily regulates the transcriptional activation of *OsWOX11*.

Ethylene, the smallest plant hormone, rapidly spreads throughout the plant and plays an important role in modulating plant growth and development (Johnson and Ecker 1998; Ma et al. 2013; Yang et al. 2015; Feng et al. 2017). Several studies have shown that ethylene interacts with other phytohormones to inhibit root elongation in rice (Ma et al. 2014; Qin et al. 2017; Huang et al. 2022; Qin et al. 2022b). In the current study, we showed that ethylene promotes crown root primordium initiation and development, leading to an increase in crown root number. Combined with previous reports (Ma et al. 2014; Qin et al. 2017; Huang et al. 2022; Qin et al. 2022b), we uncovered the dual role of ethylene in root development, namely, inhibiting root elongation and promoting crown root primordium initiation and development. Shallow rooting is advantageous for the acquisition of nutrients such as phosphorus from the topsoil, whereas deep rooting is favorable for the acquisition of water and nitrogen from the subsoil (Lynch 2013). Thus, the dual role of ethylene in the root development is an optimal root configuration in unfavorable conditions. In addition, *EIN2-OX* and *EIL1-OX* plants had more crown roots, bigger grain size, higher thousand-grain weight, shorter plants, and lower grain yield per plant compared with that of wild-type plants, possibly due to the increase of *OsWOX11* expression. Correspondingly, OsWOX11 positively regulates grain size, but its overexpression and loss-of-function lead to reduced plant height and grain yield per plant, perhaps due to its role in shoot development (Zhao et al. 2009; Cheng et al. 2018). Therefore, the precise manipulation of ethylene activity in different organs and at different stages of development is essential for achieving optimal growth and higher grain production in rice.

Soil compaction is a serious global problem that impacts crop yields by limiting soil exploration and resource capture by plant roots (Correa et al. 2019; Pandey and Bennett 2023). In agricultural soils, soil compaction occurs more easily in wet soils (Lipiec and Stepniewski 1995). Rice is a semi-aquatic plant that grows in a water-saturated environment for most of its life cycle; this means that rice roots are more susceptible to soil compaction. Previous studies have shown that soil compaction restricts ethylene diffusion to inhibit root elongation (Pandey et al. 2021; Huang et al. 2022). In the present study, we showed that soil compaction stimulates crown root primordium initiation and development, leading to an increase in crown root number in rice. Furthermore, soil compaction promotes OsEIL1 protein accumulation in roots, which further activates the expression of *OsWOX11* to modulate crown root primordium initiation and development. Mutants *ein2*, *eil1*, and *oswox11* exhibited a reduced response to ethylene and soil compaction. These findings indicate that the OsEIL1–OsWOX11 module is required for soil compaction-stimulated crown root development in rice seedlings. Therefore, our results reveal a mechanism modulating the development of the root systems of rice seedlings in the soil, which should facilitate breeding new rice cultivars with optimized root architecture.

Maintaining stable high yields under fluctuating environmental conditions is a long-standing goal of crop improvement but is challenging due to internal tradeoff mechanisms (Deng et al. 2017; Takatsuji 2017). Many of these tradeoffs are caused by gene pleiotropy (Uauy et al. 2006; Miura et al. 2010). Reducing gene pleiotropy by identifying the favorable alleles will facilitate crop breeding to overcome the tradeoff effects (Guo et al. 2021; Song et al. 2022). In the present study, soil compaction promotes ethylene production and OsEIL1 protein accumulation in roots, constitutive overexpressing *OsEIN2/OsEIL1* resulted in increased crown root number and inhibited root elongation. Moreover, grain length, grain width, and thousand-grain weight in mature plants were increased, whereas the total grain weight per plant was decreased in *EIN2-OX*, *EIL1-OX*, and *OsWOX11-OX* plants. Thus, precise manipulation of ethylene actions could contribute to improvement of root development and crop yield, and the positive role of ethylene on grain size is conducive to reducing the yield penalties due to compacted soil conditions. Further studies should focus on the identification of favorable alleles of *OsEIN2*, *OsEIL1*, and *OsWOX11*, ultimately balancing root development and crop yield.

Taken together, our results support a model in which ethylene fine-tunes crown root development primarily by stimulating *OsWOX11* expression in soil (Fig. 7C). In paddy fields, the top tillage layer is ∼15 to 20 cm thick. As the roots penetrate the soil, the higher impedance they encounter stimulates ethylene biosynthesis and the compaction of the soil pores restricts ethylene diffusion, leading to the accumulation of OsEIL1 levels in roots. OsEIL1, in turn, activates the expression of *OsWOX11* to promote crown root primordium initiation and development, thereby increasing crown root number. Our findings provide valuable insights into the regulatory role of ethylene in modulating crown root development in response to soil compaction. This understanding is vital for the establishment of robust root systems, enabling efficient resource foraging in the soil and enhancing the plant's chances of survival under diverse soil conditions.

## Materials and methods

### Plant materials and growth conditions

Rice (*Oryza sativa* ssp. *japonica*) varieties Nipponbare (Nip), Hwayoung (HY), and Zhonghua 11 (ZH11) were used as wild-type plants and background for genetic transformation in this study. The rice knockout mutants *ein2*, *eil1*, and *oswox11-4* and *OsEIN2* (*EIN2-OX*) and *OsEIL1* (*EIL1-OX*, *OsEIL1-myc*)-overexpressing transgenic lines were described previously (Zhao et al. 2009; Ma et al. 2013; Yang et al. 2015; Qin et al. 2017). To mimic soil compaction, rice seeds were surface-sterilized and planted on half-strength Murashige and Skoog medium (1/2 MS; pH 5.8) with 0.2% (w/v), 0.5% (w/v), or 1.0% (w/v) agar. Ethylene treatment was performed as previously described (Yang et al. 2015). Briefly, germinated rice seeds were placed on a stainless-steel sieve and watered with Yoshida's culture solution (Cui et al. 2015). Ethylene gas was injected into the boxes using a syringe. The seeds were placed in a growth chamber under a 14-h light (30°C)/10-h dark (25°C) photoperiod, with a light intensity of ∼150 *μ*mol/m^2^/s (white light) and 60% relative humidity for 10 d, and then crown root number was counted. For material propagation, crossing, and investigation of agronomic traits, rice plants were grown in the experimental field of the Chinese Academy of Agricultural Sciences in Beijing from May to October of each year.

### Generation of transgenic rice plants

To generate overexpression transgenic plants, the *OsWOX11* coding sequence was cloned into plant expression vector pCAMBIA1307 (Nco I and Spe I digestion) under the control of the *CaMV 35S* promoter. For knockout vector of *OsWOX11*, the knockout target was screened by the CRISPR Primer Designer (Yan et al. 2015). The CRISPR/Cas9 plasmids were generated by inserting the targets into pHUN4c12 (Bsa I digestion) vector backbone, and the recombinant vector was transformed into Agrobacterium (*Agrobacterium tumefaciens*) strain EHA105-pSOUP for rice transformation as previous described (Xu et al. 2017). All constructs were introduced separately into Nipponbare or Zhonghua 11 by Agrobacterium-mediated transformation. The primers used for plasmid construction are listed in Supplementary Table S1.

### Soil compaction experiments

The soil compaction experiment was performed as previously described with slight modification (Qin et al. 2022a). Briefly, nutrient soil was passed through a sieve with a 2 mm mesh size and then mixed with vermiculite (v/v = 2:1). Subsequently, the soil was lightly sprayed with sterilized water until the moisture content of the damp soil reached 80% (80 mL sterilized water per 100 g soil), mixed thoroughly and stored in dark for 3 to 4 d to room temperature to equilibrate. For uncompacted treatment, the wet soil was placed in a glass cylinder (20 cm × 6 cm) until the height of soil column reaches 15 cm. For soil compaction, 2.5 times volume of uncompacted soil was compressed to a height of 15 cm. Germinated rice seeds were placed on the soil surface and covered with a 2 cm top layer of wet soil, and spray water daily to keep the soil moist. Seedlings were grown for 10 d in a growth chamber under a 14 h light (30°C)/10 h dark (25°C) photoperiod, with a light intensity of ∼150 *μ*mol/m^2^/s (white light) and 60% relative humidity. Root and shoot phenotypes were observed by flushing the soil with tap water, and crown root number, shoot length, and fresh weight of shoots were counted.

### Reverse transcription quantitative PCR (RT-qPCR)

Total RNA was extracted from the samples using an Ultrapure RNA Kit (CWBIO, CW0581M). The first-strand cDNA was synthesized with 2 *μ*g of total RNA by using HiScript II Q RT SuperMix (Vazyme, R223-01) according to the instructions from the supplier. RT-qPCR assays were performed on the CFX96 Real-Time System (Bio-Rad, USA) with the rice *OsActin1* gene as an internal standard to normalize gene expression. The RT-qPCR primers are listed in Supplementary Table S1.

### In situ hybridization

In situ hybridization and immunological detection were performed as previously described (Zhao et al. 2009). The *OsWOX11* probe was amplified using gene-specific primers containing T7 and SP6 sequences (see Supplementary Table S1). The PCR fragments were purified and transcribed in vitro for sense or antisense strand synthesis using a Digoxigenin RNA Labeling Kit (Roche, 11093274910).

### Histological observation

Rice tissue sections were generated as previously described (Liu et al. 2005). Stem bases of 4-d-old seedlings were fixed in FAA solution [50% (v/v) ethanol, 10% (v/v) formaldehyde, 5% (v/v) glacial acetic acid] at 4°C for at least 48 h, dehydrated, and embedded in Spurr's resin. Sections (1 *µ*m thickness) were cut with a microtome and stained with 0.5% (w/v) toluidine blue to observe crown root primordia. Images were captured under a light microscope (Zeiss, Axio Imager.A2).

### Protein isolation and immunoblot assay

Roots of 10-d-old seedlings treated with or without ethylene for 6 h were collected, and total proteins were extracted from roots in extraction buffer containing 50 mM Tris-HCl (pH 7.5), 150 mM NaCl, 5 mM EDTA, 1% (w/v) sodium deoxycholate, 1% (v/v) Triton X-100, 0.1% (v/v) SDS, 1 mM PMSF, and 1× complete protease inhibitor cocktail (Sigma-Aldrich, P9599). Lysates were centrifuged at 15,000 × *g* for 15 min at 4°C, and the supernatant was used for immunoblot assay. Anti-Actin (Abmart, M20009, 1:4,000 dilution) and anti-Flag (Sigma-Aldrich, F1804, 1:3,000 dilution) were used to detect ACTIN and OsWOX11 protein levels. Protein levels were quantified using Image Gauge V3.12 (Fujifilm). All immunoblot experiments were repeated at least three times, essentially with the same conclusions, and representative results are shown.

### Luciferase transient expression assay

The transient expression assays were performed using rice protoplasts and *N. benthamiana* leaves. The 2.0 kb *OsWOX11* promoter region was cloned into pGREENII0800 (Kpn I and BamH I digestion) vector (*ProOsWOX11:LUC*), and the effector was generated by inserting the coding sequence of *OsEIL1* into pCAMBIA1307 (Xba I and BamH I digestion) plasmid under control of the *35S* promoter (*Pro35S:OsEIL1*). The reporter plasmid (*ProOsWOX11:LUC*) and the effector plasmids (*Pro35S:OsEIL1*) were transformed into Agrobacterium strain GV3101. The cells were resuspended in infiltration buffer (10 mM MES, 0.2 mM acetosyringone, and 10 mM MgCl_2_) to a final optical density (OD_600_ nm) = 1. Equal amounts of different combined suspensions were infiltrated into the young leaves of 5-week-old *N. benthamiana* plants using a needleless syringe. After growing the plants in the dark for 12 h, the infiltrated plants were cultivated under a 16 h light/8 h dark cycle for 48 h at 24°C. Before observation, the leaves were sprayed with 100 mM luciferin (Promega, E1602) and placed in the dark for 5 min. A low-light cooled CCD imaging apparatus (iXon; Andor Technology) was used to observe the LUC activity of each sample.

To quantitatively analyze luciferase (LUC) activity, protoplasts were prepared and transfected with the corresponding constructs via polyethylene glycol-mediated transfected as previously described (Bart et al. 2006). Firefly LUC and Renilla luciferase (REN) activities were measured with a dual-luciferase reporting assay kit (Promega, E1980). LUC activity was normalized to REN activity, and the relative LUC/REN ratios were calculated. For each plasmid combination, three independent transformations were performed.

### ChIP–qPCR assay

ChIP assays were performed as previously described (Saleh et al. 2008). Approximately 2 g samples of root tissue from wild-type (cv. Nipponbare) and *OsEIL1-myc* transgenic plants were cross-linked in 1% (v/v) formaldehyde under vacuum for 30 min. Chromatin was purified from the samples and fragmented via ultrasound treatment (sonication) to a size of 200 to 500 bp, and 3% of the yield was set aside as input. The protein–DNA complex was coimmunoprecipitated with anti-myc (Abmart, 324572, 1:3,000 dilution) antibody and protein A/G beads (Millipore, 3795223) or with protein A/G beads alone for the no-antibody control. The precipitated DNA was analyzed by qPCR using primers listed in Supplementary Table S1.

### Electrophoretic mobility shift assay (EMSA)

Plasmid construction and protein purification of N-terminal OsEIL1 (amino acids 1 to 350) were performed as previously described (Qin et al. 2017). Single-stranded complementary oligonucleotide fragments containing the OsEIL1-binding elements from the *OsWOX11* promoter were synthesized and biotinylated (Sangon Biotech). Biotin end-labeled and unlabeled probes were generated by annealing of biotinylated and unlabeled complementary primer pair, respectively. EMSA was performed using the LightShift Chemiluminescent EMSA Kit (Thermo Fisher, 20148). Reaction solutions were incubated for 20 min at room temperature. The reaction products were analyzed on native polyacrylamide gels (5%, v/v) and transferred to a nylon membrane (GE, RPN303B). Following crosslinking under UV light, the biotin end-labeled DNA was detected using the Chemiluminescent Nucleic Acid Detection Module (Thermo Fisher, 89880). The oligonucleotide sequences used are listed in Supplementary Table S1.

### Transcriptome analysis

Ten-day-old Nip and *ein2* seedlings were treated with or without 10 *μ*L/L ethylene for 3 h. Roots were collected and subjected to transcriptome analysis with three biological replicates. Raw data (raw reads) were processed using Trimmomatic (Bolger et al. 2014). After removing the reads containing poly-N and the low-quality reads, clean reads were obtained and mapped to reference genome using HISAT2 (Kim et al. 2015). Gene expression levels were estimated by fragments per kilobase of transcript per million fragments mapped and genes with expression level less than 0.1 were filtered out. DEGs were identified using the DESeq R package (Anders and Huber 2013) and (FC ≥ 2, *q*-value < 0.05) was set as the threshold for significantly differential expression. DEGs in ethylene treated and untreated Nip were defined as ERGs. In *ein2* mutant, ERGs that no longer respond to ethylene (*q*-value ≥ 0.05 or −2 < FC < 2, *q*-value <0.05) or exhibit an opposite ethylene response pattern compared with Nip (induced by ethylene in Nip, repressed by ethylene in *ein2* or repressed by ethylene in Nip, induced by ethylene in *ein2*) were identified as OsEIN2-dependent ERGs, respectively. GO enrichment analysis of DEGs was performed using R based on the hyper-geometric distribution (Young et al. 2010).

### Ethylene production measurement

Ethylene emission was measured as described previously, with some modifications (Qin et al. 2019). Rice seeds (20 seeds per sample) were surface-sterilized and planted in a 250 mL uncapped vial with 1/2 MS medium. After culturing in a growth chamber under a 14-h light (30°C)/10-h dark (25°C) photoperiod, with a light intensity of ∼150 *μ*mol/m^2^/s (white light) and 60% relative humidity for 10 d, the vials were sealed with a rubber syringe cap for 12 h, and 1 mL of headspace of each vial was collected and used to analyze ethylene production with a gas chromatograph (Hitachi, Tokyo, Japan).

### Statistical analysis

Student's *t*-test was used for significant difference analysis between two samples. One-way ANOVA followed with Tukey's test (*P* < 0.05) was used for pairwise multiple comparisons. All the analyses were performed with GraphPad Prism 5 software. Data for all statistical analyses are shown in Supplementary Data Set 3.

### Accession numbers

Sequence data from this article can be found in the Rice Genome Annotation Project website (http://rice.plantbiology.msu.edu/) under the following accession numbers: *OsActin1*, LOC_Os03g50885; *OsWOX11*, LOC_Os07g48560; *OsEIL1*, LOC_Os03g20790; OsEIN2, LOC_Os07g06130; *ERF63*, LOC_Os09g11480; *RAP2.8*, LOC_Os11g05740; *ERF2*, LOC_Os06g08340; *IAA20*, LOC_Os06g07040; *SHR5*, LOC_Os08g10310; *OsRR2*, LOC_Os02g35180; *OsCKX4*, LOC_Os01g71310. The transcriptome data from this study can be found in the National Center for Biotechnology Information Sequence Read Archive (NCBI SRA) under the accession number PRJNA988911.

## Supplementary Material

koae083_Supplementary_Data

## Data Availability

The data underlying this article are available in the article and in its online supplementary material.

## Supplementary data

The following materials are available in the online version of this article.

**Supplementary Figure S1.** Soil compaction promotes crown root development and ethylene accumulation in roots.

**Supplementary Figure S2.** Disrupting ethylene signaling inhibits ethylene-induced crown root development.

**Supplementary Figure S3.** Disrupting ethylene signaling weakens soil compaction-inhibited shoot development.

**Supplementary Figure S4.** Agronomic traits of *OsEIN2* and *OsEIL1* mutants and overexpression plants.

**Supplementary Figure S5.** GO term analysis of OsEIN2-dependent ethylene-responsive genes (ERGs).

**Supplementary Figure S6.** Root traits of mutants and overexpression lines of *OsWOX11*.

**Supplementary Figure S7.** Agronomic traits analysis of mutants and overexpression lines of *OsWOX11*.

**Supplementary Figure S8.** *OsWOX11*-mediated pathway is required for ethylene-induced crown root development.

**Supplementary Figure S9.** Mutation of *OsWOX11* disrupted ethylene-induced crown root development.

**Supplementary Figure S10.** Mutation of *OsWOX11* disrupted soil compaction-inhibited shoot development.

**Supplementary Figure S11.** Ethylene-induced *OsWOX11* expression requires an intact ethylene-signaling pathway.

**Supplementary Figure S12.** The ethylene-signaling pathway mediates ethylene-regulated *OsRR2* and *OsCKX4* expression.

**Supplementary Figure S13.** Ethylene-regulated *OsRR2* and *OsCKX4* expression requires a functional *OsWOX11*.

**Supplementary Figure S14.** OsEIL1 activates the promoter activity of *OsWOX11*.

**Supplementary Figure S15.** The effects on grain size triggered by the overexpression of *OsEIL1* are suppressed in the *oswox11* mutant.

**Supplementary Figure S16.** Disrupting ethylene signaling inhibits soil compaction-induced *OsWOX11* expression.

**Supplementary Table S1.** Primers used in this study.

**Supplementary Data Set 1.** Differentially expressed genes in response to ethylene in Nip and *ein2* roots.

**Supplementary Data Set 2.** OsEIN2-dependent ethylene-regulated genes.

**Supplementary Data Set 3.** Data for all statistical analyses performed in this study.

## Dive Curated Terms

The following phenotypic, genotypic, and functional terms are of significance to the work described in this paper:

EIL1 Gramene: Os03g20790
EIL1 Araport: Os03g20790

ERF3 Gramene: Os01g58420
ERF3 Araport: Os01g58420

IAA20 Gramene: Os06g07040
IAA20 Araport: Os06g07040

ERF2 Gramene: Os06g08340
ERF2 Araport: Os06g08340

ARF1 Gramene: Os11g32110
ARF1 Araport: Os11g32110

CTR1 Gramene: AT5G03730
CTR1 Araport: AT5G03730

EIN2 Gramene: Os07g06130
EIN2 Araport: Os07g06130

SRA Gramene: Sequence Read Archive
SRA Araport: Sequence Read Archive

CRL1 Gramene: Os03g05510
CRL1 Araport: Os03g05510
