# Supplementary material for: The OsEIL1–OsWOX11 transcription factor module controls rice crown root development in response to soil compaction
Source: Plant Cell. 2024 Mar 15;36(6):2393–409. doi: 10.1093/plcell/koae083 (PMC11132869; doi:10.1093/plcell/koae083)
Supplement: koae083_Supplementary_Data [file koae083_supplementary_data.zip › tpc.24.00004Supplemental Figures and Tables.pdf]

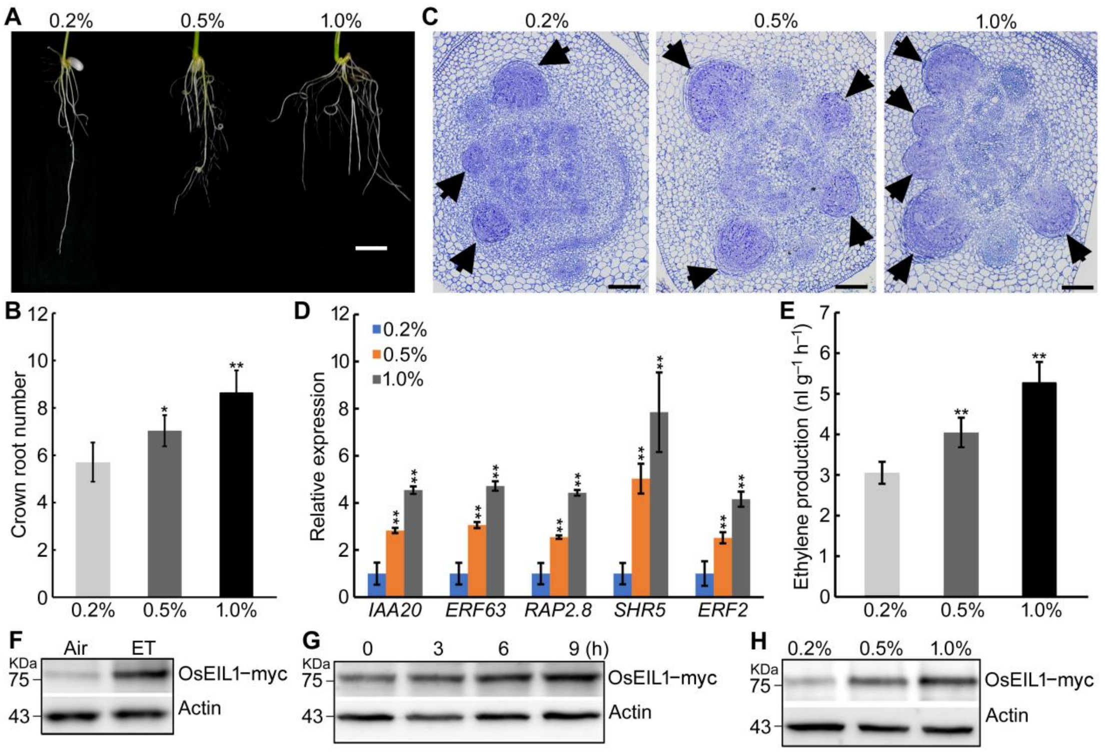

Supplemental Figure S1. Soil compaction promotes crown root development and ethylene accumulation in roots. (Supports Figure 1)
(A) Root phenotypes of 10-d-old seedlings grown on different concentrations of agar. Bar = 10 mm. (B) Crown root number of plants shown in (A). The values are means ± SD of 20 to 30 independent seedlings per sample. (C) Representative toluidine blue-stained cross sections of the stem base of 4-d-old seedlings grown on different agar concentrations. Arrows indicate crown root primordium. Bars = 100 µm. (D) Expression of ethylene-responsive genes in roots of seedlings grown on different agar concentrations. RNA was isolated from roots for qPCR. Values are mean ± SD of three replicas. (E) Ethylene production in 10-d-old seedling grown on different agar concentrations. Values are mean ± SD of three replicas. (F and G) The accumulation of OsEIL1 protein in 10-d-old seedling roots with long-term (F) and short-term (G) ethylene treatment. (H) Effects of soil compaction on OsEIL1 protein accumulation. The roots of 10-d-old OsEIL1-myc plants grown on different agar concentrations were collected for immunoblot analysis. For (B), (D) and (E), the asterisks indicate significant differences compared with 0.2% agar (*$P < 0.05$; **$P < 0.01$, Student's *t*-test).

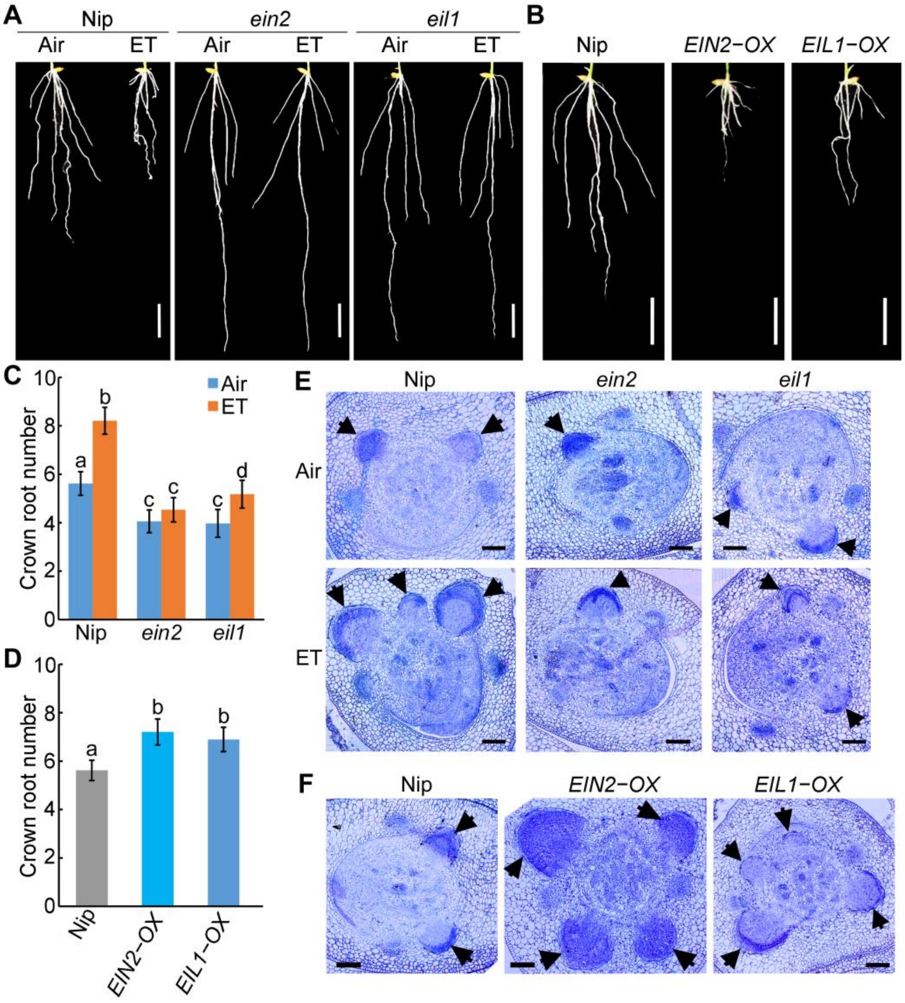

Supplemental Figure S2. Disrupting ethylene signaling inhibits ethylene-induced crown root development. (Supports Figure 1)
(A) Root phenotypes of 10-d-old Nipponbare (Nip), *ein2*, and *eil1* seedlings with or without 10 µL/L ethylene (ET) treatment. Bar = 10 mm. (B) Root phenotypes of 10-d-old Nip, *EIN2-OX* (overexpressing *OsEIN2*), and *EIL1-OX* (overexpressing *OsEIL1*) seedlings. Bar=10 mm. (C and D) Crown root number of plants shown in (A) and (B). Each column is average of 20-30 independent seedlings and bars indicate ±SD. Different letters indicate significant differences (P < 0.05, one-way ANOVA with Tukey's test). (E) Representative toluidine blue-stained cross sections of the stem base of 4-d-old Nip, *ein2*, and *eil1* seedlings with or without 10 µL/L ethylene treatment. Arrows indicate crown root primordium. Bars = 100 µm. (F) Representative toluidine blue-stained cross sections of the stem base of 4-d-old Nip, *EIN2-OX*, and *EIL1-OX* seedlings without 10 µL/L ethylene treatment. Arrows indicate crown root primordia. Bars = 100 µm.

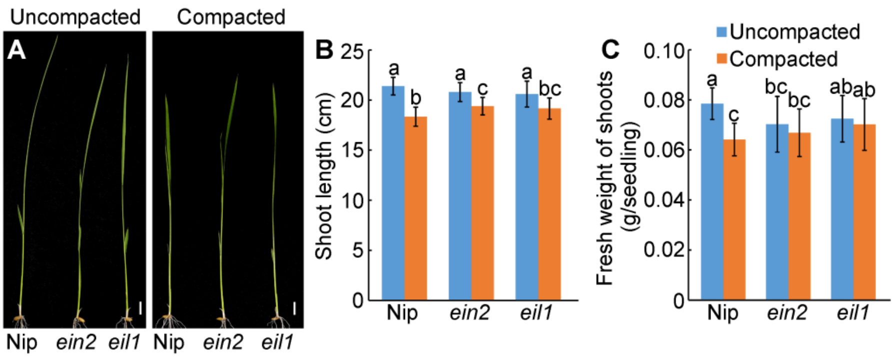

Supplemental Figure S3. Disrupting ethylene signaling weakens soil compaction-inhibited shoot development. (Supports Figure 1)
(A) Shoot phenotypes of 10-d-old Nipponbare (Nip), *ein2*, and *eil1* seedlings grown in uncompacted and compacted soil conditions. Bar = 10 mm. (B and C) Shoot length and fresh weight of shoots of plants shown in (A). Each column is average of 20-30 independent seedlings and bars indicate ± SD. Different letters indicate significant differences (*P* < 0.05, one-way ANOVA with Tukey's test).

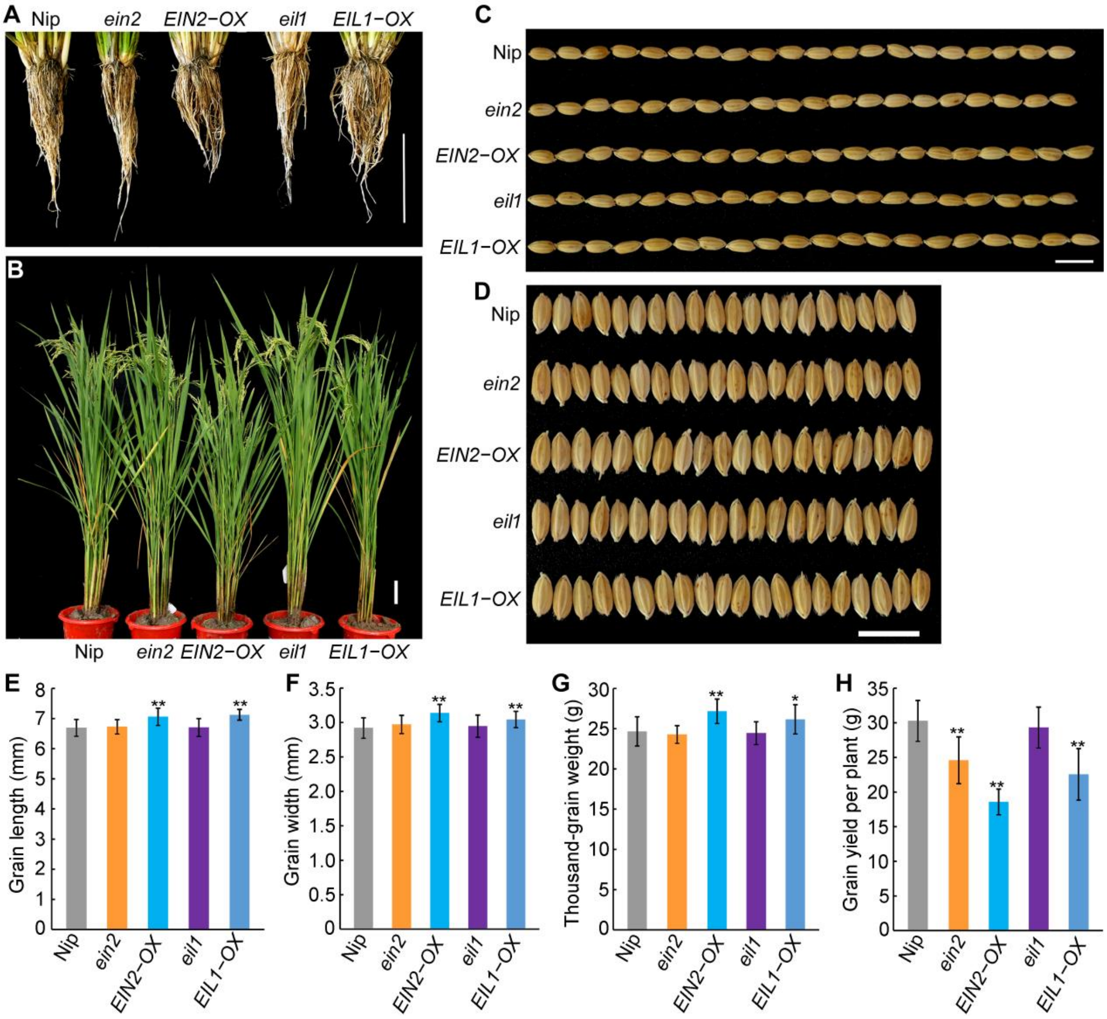

Supplemental Figure S4. Agronomic traits of *OsEIN2* and *OsEIL1* mutants and overexpression plants. (Supports Figure 1)

(A) Root phenotypes of *OsEIN2* and *OsEIL1* mutants and overexpression (OX) plants after heading. Bar = 10 cm. (B) Plant phenotypes of *OsEIN2* and *OsEIL1* mutants and overexpression plants after heading. Bar = 20 cm. (C and D) Comparison of grains of *OsEIN2* and *OsEIL1* mutants and overexpression plants. Bar = 10 mm. (E and F) Grain length (E) and grain width (F) of well-filled grains. (G) Thousand-grain weight of well-filled grains. (H) Grain yield for each plant of *OsEIN2* and *OsEIL1* mutants and overexpression plants. For (E), (F), (G) and (H), each column is average of 20 independent plants and each plant has 50–150 grains. Bars indicate ± SD. The asterisks indicate significant differences compared with Nipponbare (Nip, **P* < 0.05; ***P* < 0.01, Student's *t*-test).

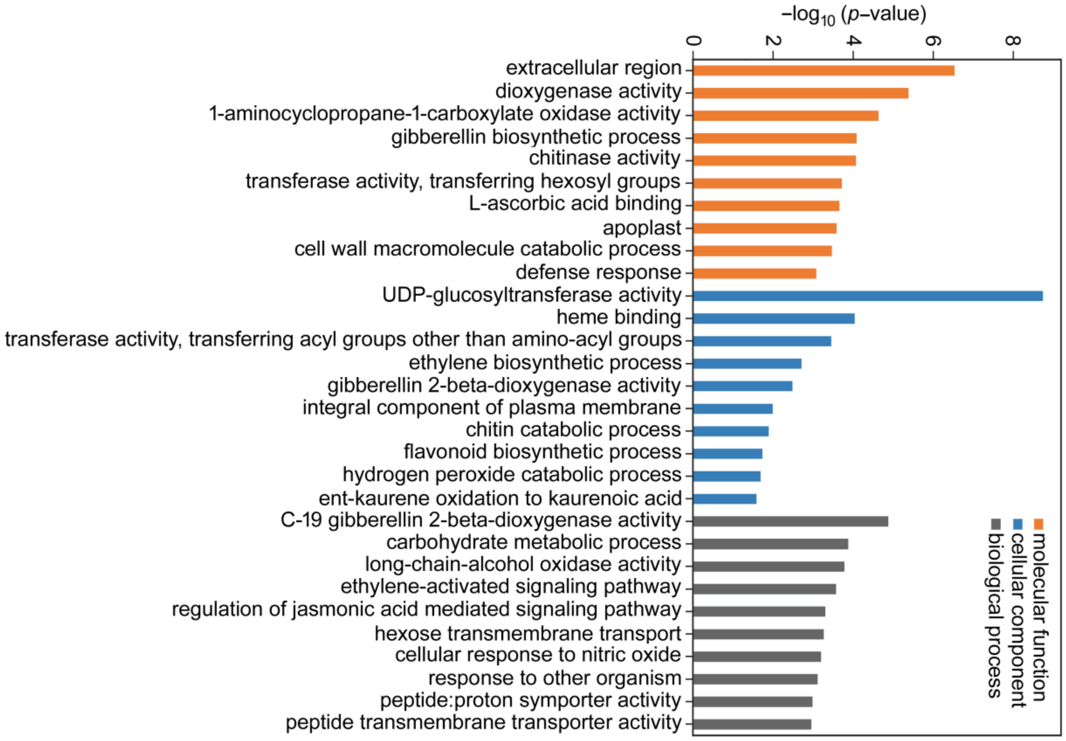

Supplemental Figure S5. GO term analysis of OsEIN2-dependent ethylene-responsive genes (ERGs). (Supports Figure 2)

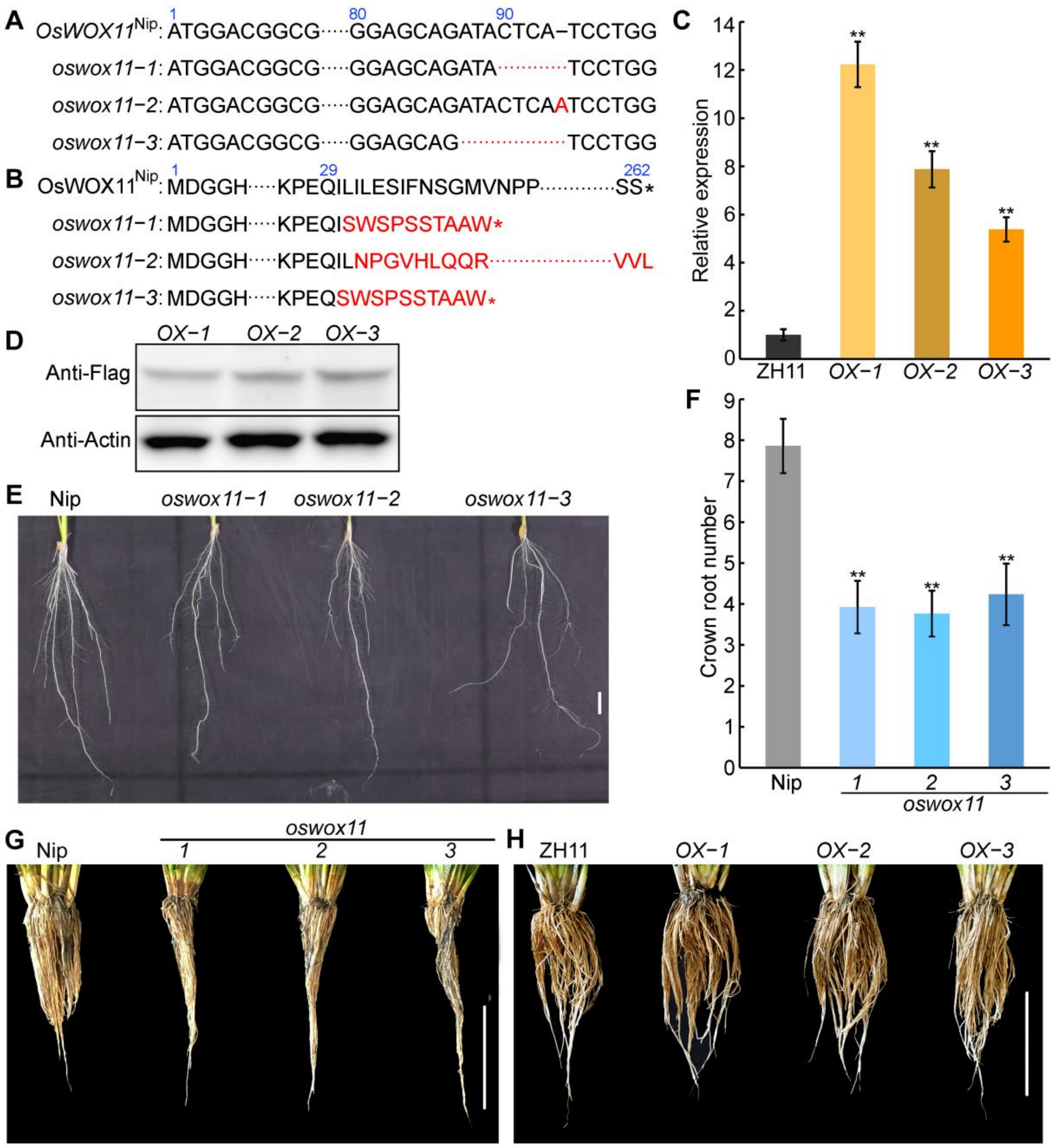

Supplemental Figure S6. Root traits of mutants and overexpression lines of *OsWOX11*. (Supports Figure 3)
(A) DNA sequences of *OsWOX11* in *oswox11* allelic mutants (*oswox11-1*, *oswox11-2* and *oswox11-3*) generated by CRISPR/Cas9. (B) Amino acid sequences of OsWOX11 in the *oswox11* allelic mutants. (C) *OsWOX11* expression in overexpression (OX) lines. Values indicate ± SD (n = 3). ** indicate significant differences by Student's *t*-test compared to Zhonghua 11 (ZH11) at $P < 0.01$. (D) The level of OsWOX11 protein in roots of *OsWOX11* overexpression lines. 10-d-old *OsWOX11* overexpression seedlings were treated with or without 10 µL/L ethylene (ET) for 6 h, and total protein was extracted from root and detected by Western Blot. (E) Root phenotypes of *oswox11* mutants at three-leaf stage. Bar = 10 mm. (F) Crown root number of plants shown in (E). Each column is average of 20-30 independent seedlings and bars indicate ± SD. ** indicate significant differences by Student's *t*-test compared to Nipponbare (Nip) at $P < 0.01$. (G and H) Root phenotypes after heading. Bar = 10 cm.

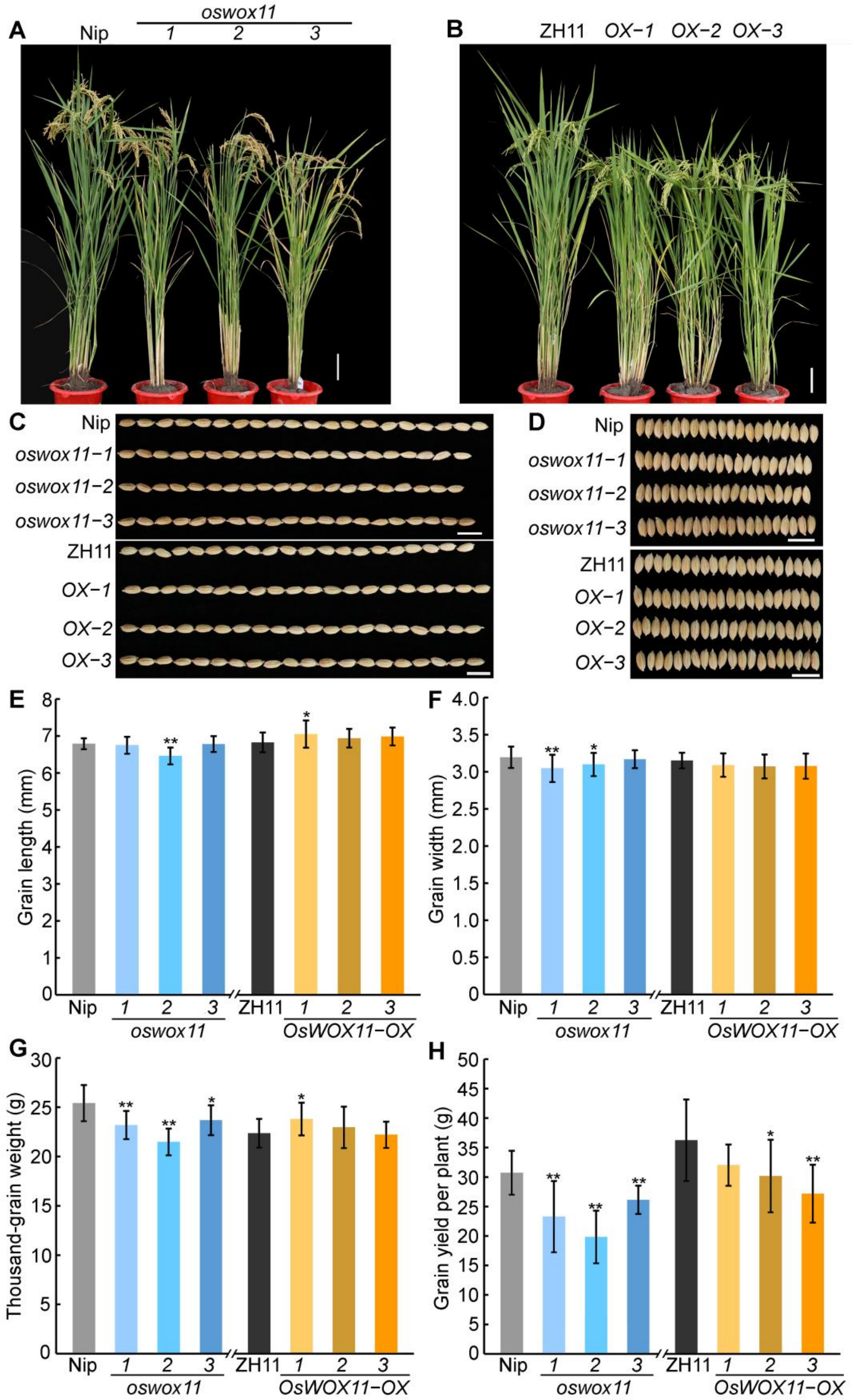

Supplemental Figure S7. Agronomic traits analysis of mutants and overexpression lines of *OsWOX11*. (Supports Figure 3)
(A and B) Plant phenotypes after heading. Bar = 20 cm. (C and D) Comparison of grains of *OsWOX11* mutants and overexpression (OX) plants. Bar = 10 mm. (E and F) Grain length (E) and grain width (F) of well-filled grains. (G) Thousand-grain weight of well-filled grains. (H) Grain yield for each plant of *OsWOX11* mutants and overexpression plants. For (E), (F), (G) and (H), each column is average of 20 independent plants and each plant has 50–150 grains. Bars indicate ± SD. The asterisks indicate significant differences compared with Nipponbare (Nip) or Zhonghua 11 (ZH11, *P < 0.05; **P < 0.01, Student’s t-test).

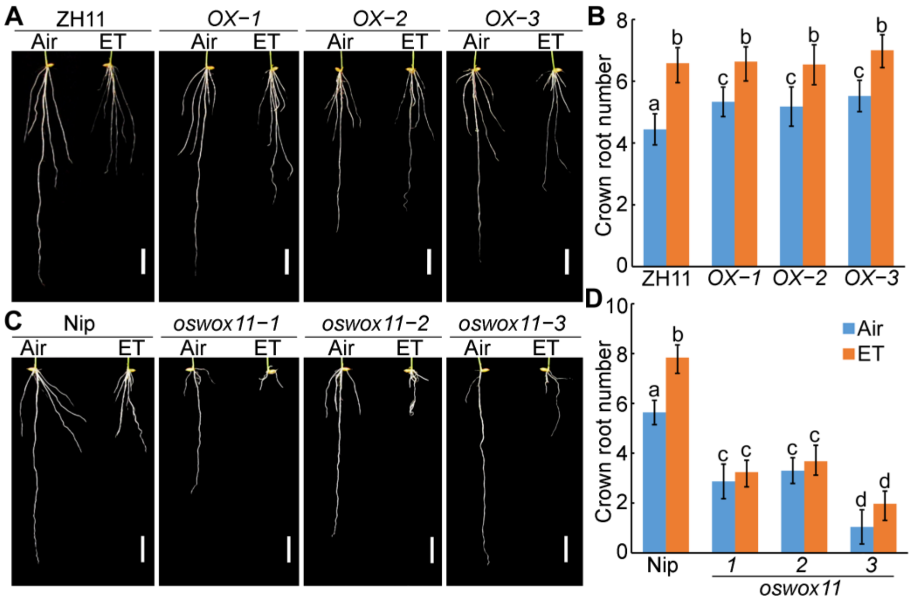

Supplemental Figure S8. *OsWOX11*-mediated pathway is required for ethylene-induced crown root development. (Supports Figure 3)
(A and C) Root phenotypes of 10-d-old *OsWOX11* overexpression (OX) lines and *oswox11* mutants with or without 10 μL/L ethylene (ET) treatment. Bar = 10 mm. (B and D) Crown root number of plants shown in (A) and (C). Values are means ± SD (n ≥ 20). Different letters indicate significant differences ($P < 0.05$, one-way ANOVA with Tukey's test). Nip represents Nipponbare and ZH11 represents Zhonghua 11.

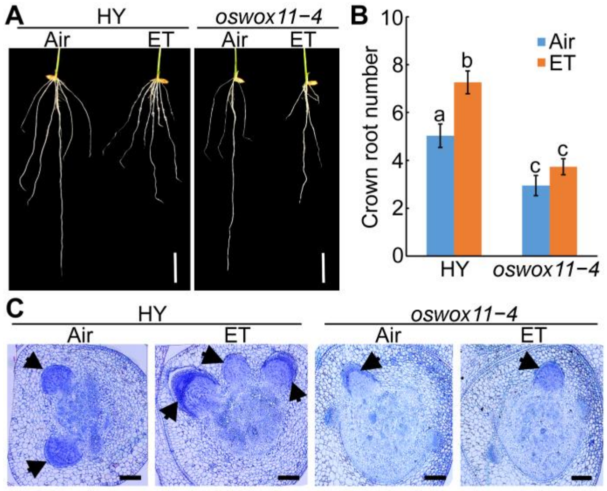

Supplemental Figure S9. Mutation of *OsWOX11* disrupted ethylene-induced crown root development. (Supports Figure 3)
(A) Root phenotypes of 10-d-old Hwayoung (HY) and *oswox11-4* mutant with or without 10 μL/L ethylene (ET) treatment. Bar = 10 mm. (B) Crown root number of plants shown in (A). Values are means ± SD (n ≥ 20). Different letters indicate significant differences ($P < 0.05$, one-way ANOVA with Tukey's test). (C) Representative toluidine blue-stained cross sections of the stem base of 4-d-old HY and *oswox11-4* seedlings with or without 10 μL/L ethylene treatment. Arrows indicate crown root primordium. Bars = 100 μm.

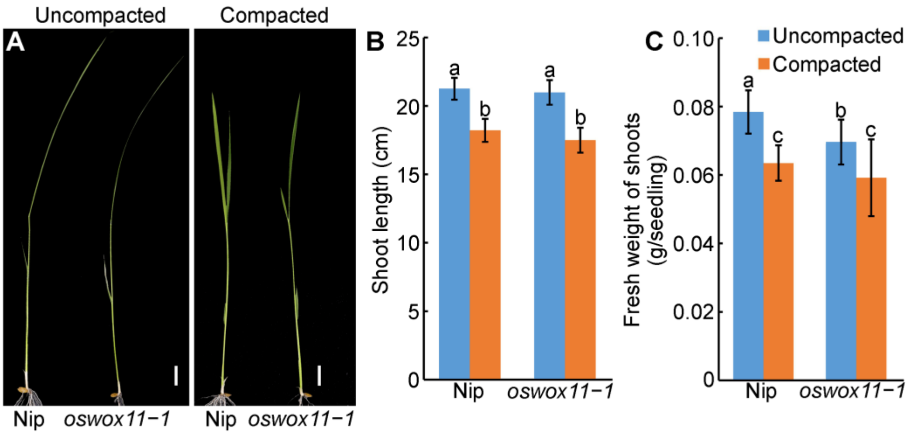

Supplemental Figure S10. Mutation of *OsWOX11* disrupted soil compaction-inhibited shoot development. (Supports Figure 4)
(A) Shoot phenotypes of 10-d-old Nipponbare (Nip) and *oswox11-1* seedlings grown in uncompacted and compacted soil conditions. Bar = 10 mm. (B and C) Shoot length and fresh weight of shoots of plants shown in (A). Each column is average of 20-30 independent seedlings and bars indicate ± SD. Different letters indicate significant differences (*P* < 0.05, one-way ANOVA with Tukey’s test).

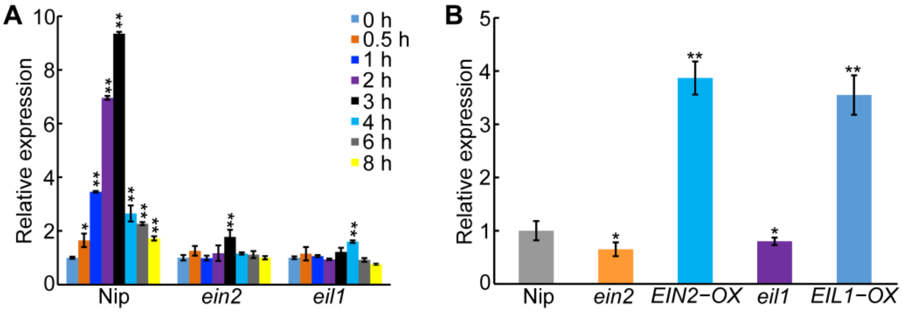

Supplemental Figure S11. Ethylene-induced *OsWOX11* expression requires an intact ethylene signaling pathway. (Supports Figure 5)

(A) Expression of *OsWOX11* in Nipponbare (Nip), *ein2*, and *eil1* seedlings. 10-d-old seedlings were treated with 10 μL/L ethylene. The RNAs from roots were isolated and used for RT-qPCR. Values are means ± SD (n = 3). * and ** indicate significant differences compared to 0 h at $P < 0.05$ and $P < 0.01$ (Student's *t*-test), respectively. (B) Expression of *OsWOX11* in 10-d-old Nip, *ein2*, *EIN2-OX* (overexpressing *OsEIN2*), *eil1*, and *EIL1-OX* (overexpressing *OsEIL1*) seedlings roots. Values are means ± SD (n = 3). * and ** indicate significant differences compared to Nip at $P < 0.05$ and $P < 0.01$ (Student's *t*-test), respectively.

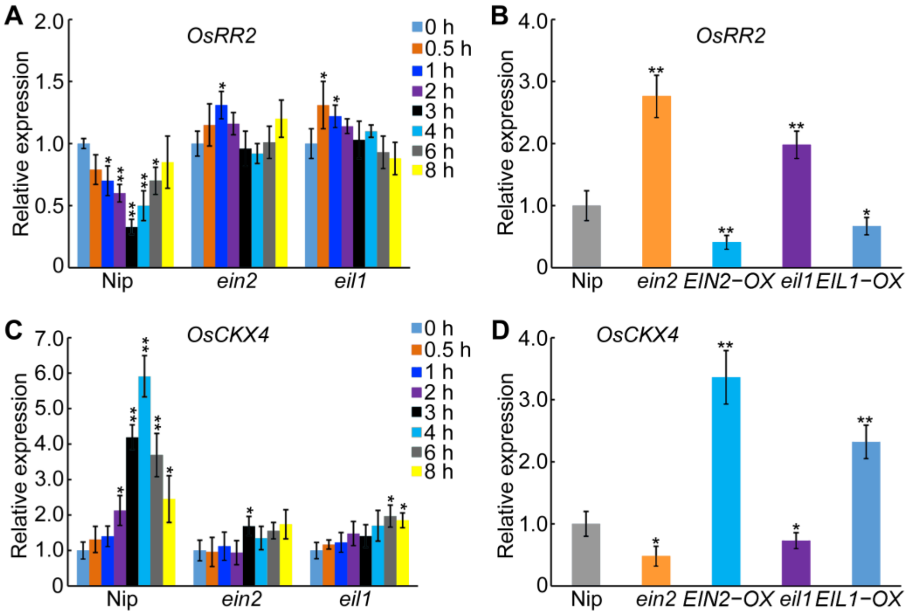

Supplemental Figure S12. The ethylene signaling pathway mediates ethylene-regulated *OsRR2* and *OsCKX4* expression. (Supports Figure 5)
(A and C) Expression of *OsRR2* (A) and *OsCKX4* (C) in Nipponbare (Nip), *ein2*, and *eil1* seedlings. 10-d-old seedlings were treated with 10 μL/L ethylene. The RNAs from roots were isolated and used for RT-qPCR. Values are means ± SD (n = 3). * and ** indicate significant differences compared to 0 h at $P < 0.05$ and $P < 0.01$ (Student's *t*-test), respectively. (B and D) Expression of *OsRR2* and *OsCKX4* in 10-d-old Nip, *ein2*, *EIN2-OX* (overexpressing *OsEIN2*), *eil1*, and *EIL1-OX* (overexpressing *OsEIL1*) seedlings roots. Values are means ± SD (n = 3). * and ** indicate significant differences compared to Nip at $P < 0.05$ and $P < 0.01$ (Student's *t*-test), respectively.

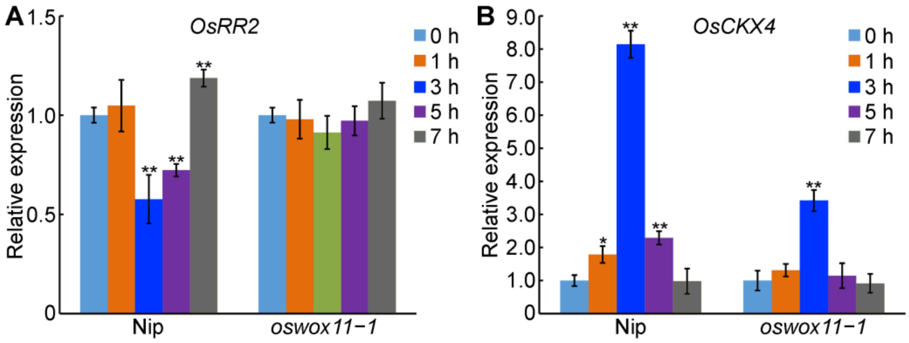

Supplemental Figure S13. Ethylene-regulated *OsRR2* and *OsCKX4* expression requires a functional *OsWOX11*. (Supports Figure 5)
(A and B) Expression of *OsRR2* (A) and *OsCKX4* (B) in Nipponbare (Nip) and *oswox11-1* seedlings. 10-d-old seedlings were treated with 10 μL/L ethylene. The RNAs from roots were isolated and used for RT-qPCR. Values are means ± SD (n = 3). * and ** indicate significant differences compared to 0 h at $P < 0.05$ and $P < 0.01$ (Student's *t*-test), respectively.

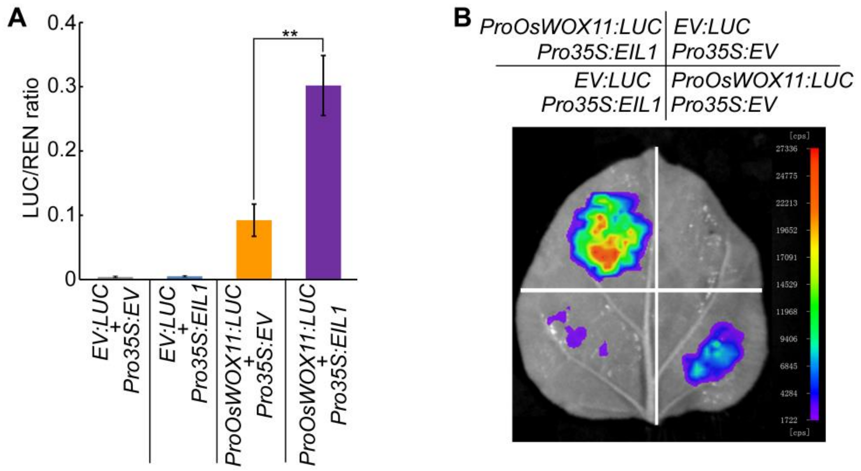

Supplemental Figure S14. OsEIL1 activates the promoter activity of *OsWOX11*. (Supports Figure 5)

OsEIL1 activates the promoter activity of *OsWOX11* in a transient expression assay in rice mesophyll protoplasts (A) or in tobacco leaves (B). 'EV' represents empty vector. Error bars represent ± SD from three independent experiments. The asterisks indicate significant difference by Student's *t*-test between compared two samples (**P < 0.01).

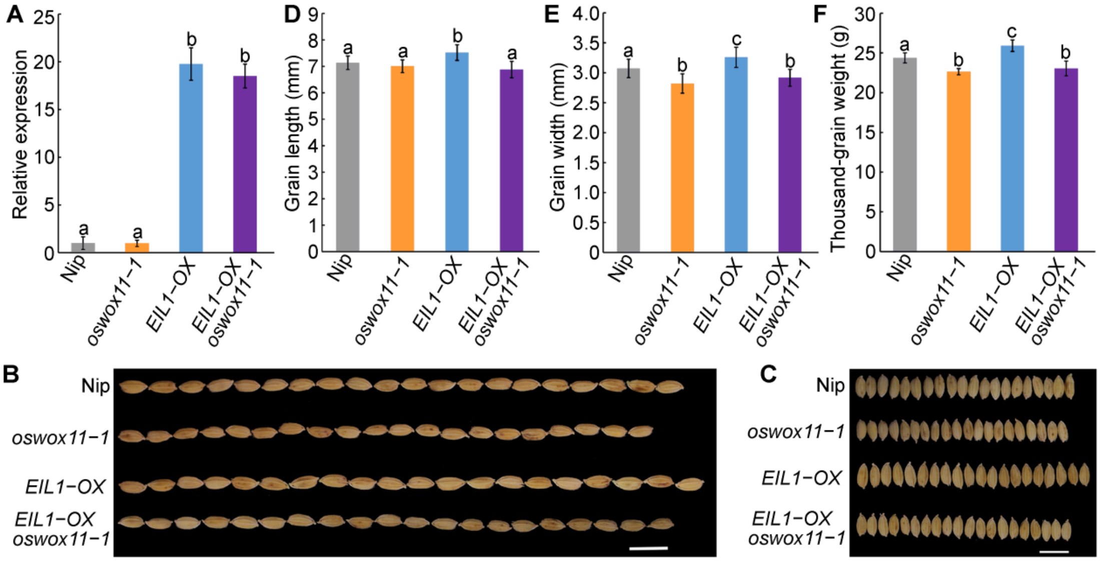

Supplemental Figure S15. The effects on grain size triggered by the overexpression of *OsEIL1* are suppressed in the *oswox11* mutant. (Supports Figure 6)

(A) Expression of *OsEIL1* in 10-d-old Nipponbare (Nip), *oswox11-1*, *EIL1-OX* (overexpressing *OsEIL1*), and *EIL1-OX oswox11-1* seedlings roots. Values are means ± SD (n = 3). (B and C) Comparison of grains of Nip, *oswox11-1*, *EIL1-OX*, and *EIL1-OX oswox11-1* plants. Bar = 10 mm. (D and E) Grain length (D) and grain width (E) of well-filled grains. (F) Thousand-grain weight of well-filled grains. For (A), (D), (E) and (F), each column is average of 20 independent plants and each plant has 50–150 grains. Bars indicate ± SD. Different letters indicate significant differences (*P* < 0.05, one-way ANOVA with Tukey's test).

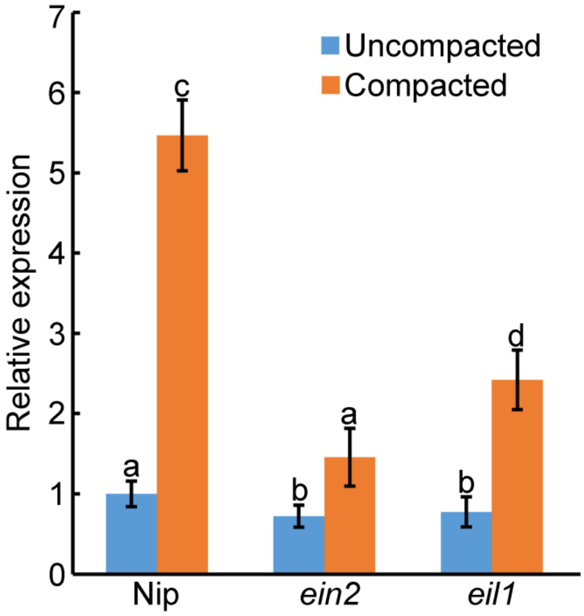

Supplemental Figure S16. Disrupting ethylene signaling inhibits soil compaction-induced *OsWOX11* expression. (Supports Figure 7)
Expression of *OsWOX11* in roots of 10-d-old Nipponbare (Nip), *ein2*, and *eil1* seedlings grown in uncompacted and compacted soil conditions. Values are means ± SD (n = 3). Different letters indicate significant differences (P < 0.05, one-way ANOVA with Tukey's test).

Supplemental Table S1. Primers used in this study.

**Primer**	**Sequence** (5'-3')
**qPCR analysis**	
OsActin1-F	GACCTTGCTGGGCGTGAT
OsActin1-R	GTCATAGTCCAGGGCGATGT
OsWOX11-F	GCATTCAACGGGTGCTCTTC
OsWOX11-R	CGATCGCGTAATTAACCGGC
OsRR2-F	ACGATCTTCTCAAAGCCATCAAG
OsRR2-R	TGAGAGGCTTAAGGATGAAATCCT
OsCKX4-F	AGAAGCAGTGGAAAGCCCACTT
OsCKX4-R	TGGAAATATTCTCTGCCCTGGA
ERF63-F	ACGTGATGGACAGCCTCCTC
ERF63-R	GGGAAGTCTGAAATGGACATG
RAP2.8-F	GAGCTCCTATGCTGCCATGT
RAP2.8-R	GGCACTATGGGGATGGAAGG
ERF2-F	GTGGACCAGATGATCGAGGAG
ERF2-R	CCAGAACTCACTGTGACCAA
IAA20-F	GCTCATACGCTGAGCTGCTCGAC
IAA20-R	CACGTCTCCGACCTGCATCCAG
SHR5-F	AATACCAGCTATGTTACCAGCC
SHR5-R	CACCATTACAAATTACAAGGAGC
OsEIL1-F	ACAATGCCACGATCATGGAG
OsEIL1-R	TCAGTAGTACCAATTCGAGC
***In situ* hybridization**	
OsWOX11-F	TAATACGACTCACTATAGGGAGAGAGTTGAGCGATTCGTCGATTG
OsWOX11-R	ATTTAGGTGACACTATAGAAGAGAGATCGAGAACGGGATACATAC
**To generate the *OsWOX11* overexpression vectors**	
OsWOX11-OX-F	CCATGGATGGACGGCGGCCACAGCC
OsWOX11-OX-R	ACTAGTAGACGACCTCGTGACCAGGAA
**CRIPSR/Cas9-mediated gene editing of *OsWOX11***	
OsWOX11-cas9-F	GGCAGCCGGAGCAGATACTCATCC
OsWOX11-cas9-R	AAACGGATGAGTATCTGCTCCGGC
**For dual-luciferase assays**	
35S-OsEIL1-F	TCTAGAATGGGAGGTGGTCTGGTGAT
35S-OsEIL1-R	GGATCCTTCCAATTCCTCAAACTCCGA
OsWOX11p-LUC-F	GGTACCCCCAATCAAATGCTCTGCC
OsWOX11p-LUC-R	GGATCCCGCCACTAGCTAGCTGCCTTGTTC
**For ChIP-qPCR**	
OsWOX11-P1F	CATGTGTTCCCCGTTCCAAG
OsWOX11-P1R	ATAGAGGATAGCCACGTAGT
OsWOX11-P2F	ACTACGTGGCTATCCTCTAT
OsWOX11-P2R	GATATGATATGTACAATGCC
OsWOX11-P3F	GGCATTGTACATATCATATC

**Primer**	**Sequence** (5'-3')
OsWOX11-P3R	ACAGTCTGAAACCCGCTTC
OsWOX11-P4F	GAAGCGGGTTTCAGACTGT
OsWOX11-P4R	TATTTAGGATGTGTGTGATG
OsWOX11-P5F	CATCACACACATCCTAAATA
OsWOX11-P5R	AGAAGTGTGTTGTTGTTAAT
**For EMSA**	
OsWOX11-Probe 1	ACAAATACTCACCGTACATGTACGGACTCACTCATATCCC
OsWOX11-Probe 2	ATACTAGGGATATTCCGATTAATGTACACGTGCCTTCAATTATA
OsWOX11-Probe 3	GAAAATGACATATACAGTACATCATGTACTCCAACTAATAATTAACAACA
OsEIL1-N-GST-F	GGATCCATGGGAGGTGGTCTGGTGAT
OsEIL1-N-GST-R	GCGGCCGCGCCGCCGGTAGGTGCCGGA
