# Supplementary material for: The OsEIL1–OsWOX11 transcription factor module controls rice crown root development in response to soil compaction
Source: Plant Cell. 2024 Mar 15;36(6):2393–409. doi: 10.1093/plcell/koae083 (PMC11132869; doi:10.1093/plcell/koae083)
Supplement: koae083_Supplementary_Data [file tpc.00004.2024-s07.pdf › tpc.00004.2024-s07.pdf]

THE PLANT CELL

OsEIL1-OsWOX11 module is required for crown root development in response to soil compaction

Yuxiang Li, Juan Wang, Yadi Gao, Bipin Pandey, Lucas Leon Peralta Ogorek, Yu Zhao, Ruidang Quan, Zihan Zhao, Lei Jiang, Rongfeng Huang, and Hua Qin

Review Timeline:

Submission Date:	17-Jul-2023
Editorial Decision:	20-Aug-2023
Resubmission Received:	08-Jan-2024
Editorial Decision:	01-Feb-2024
Revision Received:	20-Feb-2024
Accepted:	21-Feb-2024

Dr. Hua Qin
Chinese Academy of Agricultural Sciences Biotechnology Research Institute
Beijing
China

Dear Dr. Qin:

Thank you for choosing to send your manuscript entitled "Ethylene activates OsWOX11 transcription to stimulate crown root development in response to soil compaction" for consideration at The Plant Cell. Your submission has been evaluated by members of the editorial board as well as expert reviewers in your field, and we regret to inform you that we are not able to recommend the publication of this manuscript in its current form. We have had input from multiple scientists and we have solicited post-review comments.

That said, we would be willing to re-consider a new manuscript that fully addressed the concerns raised during this review process, and detailed below.

It will be important to convince the editors and reviewers that the study adds significant new understanding(s) of mechanisms or processes, or otherwise represents a significant advance in the field, and that the major claims made are fully justified by the data presented. This includes careful consideration and explanation of the various controls used in experiments, the extent and manner of replication, and the statistical analyses used. Sampling methods and the nature of "biological replicates" should be described precisely (i.e. different plants, parts of plants, pooled tissue, independent pools of tissue, sampled at different times, etc.), along with a clear description of and rationale for any statistical analyses conducted. The reader should know exactly what was sampled; what forms the basis of the calculation of any means and other statistical variables and parameters reported. This is also necessary to ensure that proper statistical analysis was conducted.

The Plant Cell now requires authors to complete and submit an author revisions checklist upon submission of a revised manuscript. The aim of the checklist is to aid authors in preparing a high-quality manuscript, facilitate the review and assessment of revised manuscripts, and help to ensure that journal standards are maintained across the board. If your manuscript is accepted, the completed checklist will be published as supplemental material attached to the article online. Please download a copy of the checklist (a fillable PDF form) at this link, for submission with your revised manuscript: https://tpc.msubmit.net/html/Author_Revisions_Checklist.pdf.

Note also that supplemental materials should be restricted to large datasets and tables, presentation of replicates, and validation of reagents, methods, or genotypes. Any data that are used to support the main claims must be in the main manuscript. Supplemental figure legends must indicate what figure in the main manuscript is supported by the supplemental data presented.

We thank you for your interest in and support of The Plant Cell. We hope we will be able to render a more positive decision on future work.

Sincerely,

The Plant Cell Board of Editors

-- Reviewer comments:

Reviewer #1 (Comments for the Author):

The authors revealed that compacted soil can promote ethylene production, and subsequently regulate WOX11 expression through the EIN2-EIL1 signaling module, and finally regulate CK signaling, which affects crown root initiation. First, authors showed that ethylene acts as a key signal that stimulates crown root development in response to soil compaction. They demonstrate that compacted soil promotes ethylene production and the accumulation of ETHYLENE INSENSITIVE 3-LIKE 1 (OsEIL1) in rice roots, stimulating crown root primordia initiation and development, thereby increasing crown root number in lower stem nodes. Then, through transcriptome profiling and molecular analyses, they reveal that OsEIL1 directly activates the expression of WUSCHEL-RELATED HOMEOBOX 11 (OsWOX11), an activator of crown root emergence and growth, and that mutations of OsWOX11 delay crown root development thus impairing the response to ethylene and soil compaction. Moreover, both OsEIL1 and OsWOX11 have been shown to positively affect grain size, and OsWOX11 transcriptionally functions downstream of OsEIL1. It is an interesting study, I have several comments on this manuscript:

Q1. Line 37-38: soil compaction promotes OsEIL1 accumulation; Line 45: OsEIL1 positively affects grain size. So does soil compaction contribute to grain size? If compacted soil were good for crop yields, then farmers could compress the soil tightly, but this is obviously not the case. It is suggested that the authors revise the corresponding expressions to avoid misconceptions.

Q2. Line 158-160: Similar to those of soil compaction, increasing agar concentration also increased the crown root number in Nip, but had no effect on ein2 and eil1 mutants. Line 165: soil compaction stimulates crown root development partially depending on ethylene signaling pathway. Since the ability of ethylene to promote crown root production is completely lost in ein2 and eil1, why do the authors conclude that it is partly dependent on ethylene signaling?

Q3. From the results of this study, in fact, the authors seem to have overlooked the fact that compacted soil and ethylene lead to a reduction in crown root length while increasing the number of crown roots. Therefore, the authors should not blindly emphasize the relationship between ethylene and the number of crown roots, but neglect to explain the fact that the development or elongation of crown roots are inhibited.

Q4. Line 83-85: Soil compaction is a serious global problem as it causes inadequate rooting and substantial reduction in crop yield (Hamza and Anderson, 2005; Correa et al., 2019; Schneider et al., 2021). Line 169-170: Importantly, grain length and grain width in mature plants were increased in EIN2-OX and EIL1-OX plants.
So, how to understand the relationship between soil compaction, ethylene, and crop yield? It is suggested that the authors examine the possibility that the degree of soil compaction may produce different concentrations of ethylene, while low concentrations of ethylene may promote crown root development and crop yield, while high concentrations have the opposite effect. Otherwise, it is impossible to explain why ethylene both boosts and inhibits crop yields.

Q5. Another interesting question is whether more crown roots necessarily contribute to increased crop yields, especially in compacted soil environments. Why do authors refer to increased yield phenotypes when describing EIN2, EIL1, and WOX11?

Q6. Line 378-380: we uncover the dual role of ethylene in root development, namely, inhibiting root elongation and promoting crown root primordium initiation and development.
According to the results of the authors, ethylene signaling (EIN2 and EIL1) obviously significantly promotes crop yield, so crown roots rather than primary root are the core factor determining crop yield ? Or is this an optimal root configuration for compacted soil?

Q7 It seems that the authors try to explain the seemingly contradictory facts of ethylene inhibiting root elongation, promoting crown root production, and promoting/inhibiting crop yield with the dose effect of ethylene. It is a bit difficult to understand why ethylene produced in response to soil compaction can boost crop yields.

Reviewer #2 (Comments for the Author):

In this manuscript, the authors investigated the root development of rice in response to compacted soil. They showed that compacted soil stimulates ethylene biosynthesis and stabilizes the accumulation of OsEIL1 proteins. By using mutants and transgenic lines, the authors showed that ethylene signaling promotes crown root development and is required for compacted soil-stimulated crown root development. Through transcriptome analysis, the authors found that OsWOX11 is involved in ethylene-promoted crown root development. The authors generated mutants and overexpressing lines of OsWOX11 and showed that OsWOX11 indeed promotes crown root development. Moreover, OsEIL1 directly binds to the promoter of OsWOX11 and activates the transcription of OsWOX11. The authors proposed a OsEIL1-OsWOX11 module in mediating compacted soil-regulated crown root development. Overall, this study is straightforward and the findings are interesting. I have several suggestions or concerns about it.

1. It has been well documented that ethylene biosynthesis is induced by soil impedance and compacted soil conditions. This study further found that ethylene signaling mediates the compacted soil-promoted crown root development. What are the biological meanings of crown root development in response to compacted soil? Are there any growth defects for ethylene signaling mutants and OsWOX11 mutants when grown in compacted soil?

2. The transcriptome analysis revealed that thousands of genes were regulated by ethylene and the authors proposed that it is the single gene of OsWOX11 in mediating compacted soil-promoted crown root development. Are there any other ethylene-regulated and root-expressed genes? What are the functions of those genes?

3. The authors proposed that the transcriptional activation of OsWOX11 by ethylene-OsEIL1 mediates the ethylene-induced crown root development. However, in Supplemental Figure 5, the crown root development of OsWOX11 overexpression lines, which the transcription levels are not regulated by ethylene, is still induced by ethylene. These results indicate other regulations

or other downstream genes rather than OsWOX11 transcriptional regulation in mediating ethylene-induced crown root development.

4. Soil impedance-induced ethylene biosynthesis was firstly reported in the paper (PNAS, 2014, 111:3913-20; doi: 10.1073/pnas.1402491111). The references for Putative EIN3-binding sites (ATGTA/TACAT) are also not indicated.

5. The authors proposed that OsEIL1 activates OsWOX11 gene expression in mediating compacted soil-induced crown root development. The gene expression levels of OsWOX11 in WT and ethylene signaling mutants under compacted soil conditions should be examined.

Reviewer #3 (Comments for the Author):

In this manuscript, the authors propose a mechanistic model where ethylene and soil compaction-triggered ethylene promote crown root formation in rice through the activation of OsWOX11. Although the data presented are mostly consistent with the proposed mechanism, in my opinion, the causal link between the ethylene-induced OsWOX11 and the promotion of crown root formation has not formally been proven. As I elaborate below, some additional experiments should be performed to test the authors' hypothesis further.

From the results displayed in Figure 2, it seems that in the ein2 mutant there are many genes regulated by ethylene that were not affected by this hormone in the wild-type plant. This needs to be explained. Similarly, I do not understand the diagram in Figure 2C diagram. The EIN2-dependent ethylene-regulated genes should be a subset of all the ethylene-regulated genes. However, the set of EIN2-dependent ERGs is much bigger than that of the ethylene-regulated genes.

I don't think the GO analysis in Figure 2D is very helpful regarding the main topic of the paper. Perhaps this should be moved to supplemental information.

The functional category of genes associated with lateral root formation is not listed. Was this functional category not enriched?

All 49 genes selected and displayed in Figure 2E are regulated by ethylene and involved in root development. Why was OsWOX11 chosen?

The oswox11-c2 mutation results in a frameshift and, I assume, a premature stop codon. This is not mentioned in the text or shown in Supplemental Figure 4B.

There is no correlation between the OSWOX11 expression levels and the number of crown roots. This needs to be discussed. Similarly, although the c1 and c3 mutant alleles are practically identical (just one aa different), the number of crown roots is significantly different. This also needs to be explained.

The fact that ethylene regulates OsWOX11 in an EIN2- and EIL1-dependent manner does not mean that the ethylene effects on the number of crown roots are mediated by an increase in the levels of OsWOX11. In fact, the observation that ethylene has a similar effect on the number of crown roots in wild type and OsWOX11 overexpression argues against the idea that the ethylene effect on crown roots is due to an increase in the levels of OsWOX11. In the OsWOX11 overexpression lines, the transcript levels are 12-fold higher (Figure S4C) than in the wild type but only slightly increase the number of crown roots (Figure S5B). On the other hand, ethylene treatment has a similar or lower effect on the transcript levels of OsWOX11 (Figure S7A) but a much greater effect on the number of crown roots (Figure S5B). I think the results indicate that the ethylene effects on crown roots require a functional OsWOX11 but do not support the model where this ethylene effect is simply mediated by the transcriptional activation of OsWOX11 by ethylene.

Related to the previous point, to test the model where ethylene-mediated upregulation of OsWOX11 is responsible for the observed increase in the expression of OsCKX4 and the reduction in the levels of OsRR2, the authors should check whether or not the ethylene effects on the expression of these two genes are blocked in the oswox11 mutants.

As indicated before, the fact that ethylene has a significant effect in the OsWOX11 overexpression lines suggests that the ethylene effect is not simply due to an increase in the transcription of OsWOX11. To shed further light on the validity of the presented model, the authors should test the effects of the oseil1 and/or osein2 mutations on the number of crown roots in the OsWOX11 overexpression lines. The expectation, according to the authors' model, is that the mutations in oseil1 and osein2 should have no effect on the number of crown roots present in the OsWOX11 overexpression lines. On the other hand, an effect of the oseil1 or osein2 mutants would suggest a more complex scenario where ethylene may affect the number of crown roots by a mechanism other than the transcriptional activation of OsWOX11. In this same line of thought, the analysis of the ethylene effects on the transcript levels of OsRR2 and OsCKX4 in the oswox11 mutant should also help to better define the mechanism by which ethylene regulates crown root number.

Are the effects on grain size triggered by the overexpression of OsEIL1 suppressed in the oswox11 mutant? Grain size should be properly quantified and proper statistical methods applied.

In this work, the authors only show grain size, but not yield. Including yield results will be relevant to evaluating the potential biotechnological applications of these findings claimed by the authors.

Other minor points:

The constructs used in the dual luciferase assay should be indicated in the materials and methods section.

To further confirm that, indeed, the oswox11 mutations suppress the effects of OsEIL1 overexpression, the levels of expression of OsEIL1 in the double mutant should be examined to confirm they are similar to those of the original overexpression line as the presence of a transgene in the oswox11 may be causing silencing of the OsEIL1 transgene.

Reviewer #1 (Comments for the Author):

The authors revealed that compacted soil can promote ethylene production, and subsequently regulate WOX11 expression through the EIN2-EIL1 signaling module, and finally regulate CK signaling, which affects crown root initiation. First, authors showed that ethylene acts as a key signal that stimulates crown root development in response to soil compaction. They demonstrate that compacted soil promotes ethylene production and the accumulation of ETHYLENE INSENSITIVE 3-LIKE 1 (OsEIL1) in rice roots, stimulating crown root primordia initiation and development, thereby increasing crown root number in lower stem nodes. Then, through transcriptome profiling and molecular analyses, they reveal that OsEIL1 directly activates the expression of WUSCHEL-RELATED HOMEOBOX 11 (OsWOX11), an activator of crown root emergence and growth, and that mutations of OsWOX11 delay crown root development thus impairing the response to ethylene and soil compaction. Moreover, both OsEIL1 and OsWOX11 have been shown to positively affect grain size, and OsWOX11 transcriptionally functions downstream of OsEIL1. It is an interesting study, I have several comments on this manuscript:

Response: We appreciate reviewer's positive comments for the quality of the manuscript.

Q1. Line 37-38: soil compaction promotes OsEIL1 accumulation; Line 45: OsEIL1 positively affects grain size. So does soil compaction contribute to grain size? If compacted soil were good for crop yields, then farmers could compress the soil tightly, but this is obviously not the case. It is suggested that the authors revise the corresponding expressions to avoid misconceptions.

Response: Thanks for your suggestions. Previous studies showed that alteration of ethylene signaling pathway affects rice grain filling (Panda et al., 2016; Yin et al., 2017). Our observations also showed that the grain length, grain width, and thousand-grain weight were increased, whereas the total grain weight per plant were decreased in *EIN2-OX* and *EIL1-OX* plants, indicating that alteration of ethylene

signaling pathway is not beneficial for net rice yields due to its negative effect on grain filling. Thus, although soil compaction promotes OsEIL1 accumulation, it does not translate to higher yield in compacted soil conditions due to poor grain filling. The positive role of OsEIL1 on grain size might be to reduce the yield penalties under adverse conditions. We have added these results and revised the corresponding expressions in the revised manuscript.

Q2. Line 158-160: Similar to those of soil compaction, increasing agar concentration also increased the crown root number in Nip, but had no effect on ein2 and eil1 mutants. Line 165: soil compaction stimulates crown root development partially depending on ethylene signaling pathway. Since the ability of ethylene to promote crown root production is completely lost in ein2 and eil1, why do the authors conclude that it is partly dependent on ethylene signaling?

Response: We are sorry for not making this conclusion accurately. We have now corrected it to "soil compaction stimulates crown root development depending on ethylene signaling pathway" in the revised manuscript.

Q3. From the results of this study, in fact, the authors seem to have overlooked the fact that compacted soil and ethylene lead to a reduction in crown root length while increasing the number of crown roots. Therefore, the authors should not blindly emphasize the relationship between ethylene and the number of crown roots, but neglect to explain the fact that the development or elongation of crown roots are inhibited.

Response: Thanks for your important suggestions. Both crown root number and root length are important for plant growth and yield. Since soil compaction and ethylene inhibits root elongation in rice have been extensively studied (Ma et al., 2014; Yin et al., 2015; Qin et al., 2017; Pandey et al., 2021; Huang et al., 2022; Qin et al., 2022), and OsWOX11 is a key regulator of crown root development (Zhao et al., 2009; Zhao et al., 2015; Zhou et al., 2017), this study mainly focused on the role of ethylene in crown root initiation in response to soil compaction.

Q4. Line 83-85: Soil compaction is a serious global problem as it causes inadequate rooting and substantial reduction in crop yield (Hamza and Anderson, 2005; Correa et al., 2019; Schneider et al., 2021). Line 169-170: Importantly, grain length and grain width in mature plants were increased in EIN2-OX and EIL1-OX plants.

So, how to understand the relationship between soil compaction, ethylene, and crop yield? It is suggested that the authors examine the possibility that the degree of soil compaction may produce different concentrations of ethylene, while low concentrations of ethylene may promote crown root development and crop yield, while high concentrations have the opposite effect. Otherwise, it is impossible to explain why ethylene both boosts and inhibits crop yields.

Response: Thanks for your suggestion. In the present study, we demonstrate that ethylene functions as a key signal to promote crown root initiation in response to soil compaction. Combined with previous reports that ethylene inhibits root elongation (Ma et al., 2014; Yin et al., 2015; Qin et al., 2017; Qin et al., 2022), this dual roles of ethylene in root growth may have an adaptive value to compacted soil. We measured ethylene production of seedlings grown on different concentrations of agar and found that ethylene production increased with increasing agar concentrations (Supplemental Figure 1E). Correspondingly, the crown root number increased with increasing agar concentrations (Supplemental Figures 1A and 1B), implying that the degree of soil compaction produces different concentrations of ethylene, which further promotes crown root development. In addition, although grain length, grain width, and thousand-grain weight in mature plants were increased, whereas the total grain weight per plant were decreased in *EIN2-OX* and *EIL1-OX* plants. The positive role of ethylene on grain size might be to reduce the yield penalties under compacted conditions. Thus, we think the production of ethylene under soil compaction is conducive for achieving optimal growth and reduce the yield penalties in rice. We have added these results in the revised manuscript.

Q5. Another interesting question is whether more crown roots necessarily contribute

to increased crop yields, especially in compacted soil environments. Why do authors refer to increased yield phenotypes when describing EIN2, EIL1, and WOX11?

Response: Thanks for your suggestion. Crown roots are the main component of the fibrous root system in cereal crops. Modulation of crown root growth could enhance crop yields and improve the plant's ability to withstand various adversities (Uga et al., 2013; Kitomi et al., 2020). Increasing crown root number could increase crop yield without changing other agronomic traits. In the present study, our results showed that soil compaction and ethylene stimulate crown root primordium initiation and development, leading to an increase in crown root number in rice. However, the root elongation is inhibited by ethylene and soil compaction, as revealed by previous studies (Ma et al., 2014; Yin et al., 2015; Qin et al., 2017; Pandey et al., 2021; Huang et al., 2022; Qin et al., 2022). Moreover, although the grain length and grain width were increased in *EIN2-OX* and *EIL1-OX* plants, this does not mean that yield has increased. In fact, the total grain weight per plant was decreased in *EIN2-OX* and *EIL1-OX* plants. Thus, we think the effect of ethylene on crown root is an optimal root configuration to achieve optimal growth and reduce the yield penalties in response to compacted soil. We have added this information the revised manuscript.

Q6. Line 378-380: we uncover the dual role of ethylene in root development, namely, inhibiting root elongation and promoting crown root primordium initiation and development.

According to the results of the authors, ethylene signaling (EIN2 and EIL1) obviously significantly promotes crop yield, so crown roots rather than primary root are the core factor determining crop yield？ Or is this an optimal root configuration for compacted soil?

Response: Thanks for your suggestion. The primary root of rice ceases to grow after 7-10 days of rapid growth, followed by the gradual emergence and growth of the crown roots (Marcon et al., 2013; Rogers and Benfey, 2015; Qin et al., 2022). The crown roots form a major functional component of the mature root system (Kitomi et al., 2020; Shao et al., 2019; Zhao et al., 2009). Therefore, crown roots rather than

primary root are the core factor determining crop yield. Moreover, although the grain length and grain width were increased in *EIN2-OX* and *EIL1-OX* plants, this does not mean that yield has increased. In fact, the total grain weight per plant was decreased in *EIN2-OX* and *EIL1-OX* plants. Thus, the dual role of ethylene in root development is an optimal root configuration in response to compacted soil.

Q7 It seems that the authors try to explain the seemingly contradictory facts of ethylene inhibiting root elongation, promoting crown root production, and promoting/inhibiting crop yield with the dose effect of ethylene. It is a bit difficult to understand why ethylene produced in response to soil compaction can boost crop yields.

Response: We are sorry for not making this statement clear. Although grain length, grain width, and thousand-grain weight in mature plants were increased, whereas the total grain weight per plant was decreased in *EIN2-OX* and *EIL1-OX* plants. So, eventually ethylene did not promote crop yield. The positively role of ethylene on grain size might be to reduce the yield penalties under compacted conditions. We have added these results and revised the corresponding expressions in the revised manuscript.

Reviewer #2 (Comments for the Author):

In this manuscript, the authors investigated the root development of rice in response to compacted soil. They showed that compacted soil stimulates ethylene biosynthesis and stabilizes the accumulation of OsEIL1 proteins. By using mutants and transgenic lines, the authors showed that ethylene signaling promotes crown root development and is required for compacted soil-stimulated crown root development. Through transcriptome analysis, the authors found that OsWOX11 is involved in ethylene-promoted crown root development. The authors generated mutants and overexpressing lines of OsWOX11 and showed that OsWOX11 indeed promotes crown root development. Moreover, OsEIL1 directly binds to the promoter of

OsWOX11 and activates the transcription of OsWOX11. The authors proposed a OsEIL1-OsWOX11 module in mediating compacted soil-regulated crown root development. Overall, this study is straightforward and the findings are interesting. I have several suggestions or concerns about it.

Response: We appreciate the positive comments by the reviewer regarding quality of the work.

1. It has been well documented that ethylene biosynthesis is induced by soil impedance and compacted soil conditions. This study further found that ethylene signaling mediates the compacted soil-promoted crown root development. What are the biological meanings of crown root development in response to compacted soil? Are there any growth defects for ethylene signaling mutants and OsWOX11 mutants when grown in compacted soil?

Response: Thank you for your constructive suggestion. Optimized root architecture of crops is an effective strategy to improve crop yields in favorable and unfavorable conditions. Ethylene and soil compaction inhibit root elongation (Ma et al., 2014; Yin et al., 2015; Qin et al., 2017; Pandey et al., 2021; Huang et al., 2022; Qin et al., 2022), more crown root number under compacted soil conditions might be an optimal root configuration to achieve optimal growth and reduce the yield penalties. Compacted soil significantly reduced the shoot length and fresh weight of shoots in Nip seedlings, with a milder phenotype in *ein2*, *eil1*, and *oswox11-c1* seedlings, indicating that the OsEIL1-OsWOX11 module is involved in soil compaction-inhibited rice seedlings development. We have added these results in the revised manuscript.

2. The transcriptome analysis revealed that thousands of genes were regulated by ethylene and the authors proposed that it is the single gene of OsWOX11 in mediating compacted soil-promoted crown root development. Are there any other ethylene-regulated and root-expressed genes? What are the functions of those genes?

Response: Thank you for your suggestion. In the present study, our results demonstrated that ethylene stimulates the initiation and development of crown root

primordia in lower stem nodes and this process is primarily mediated by the ethylene-signaling pathway. To dissect the molecular mechanisms underlying ethylene-regulated crown root development, we mainly focused on genes that are regulated by ethylene signaling components. Through transcriptome profiling analyses, we identified 49 genes associated with root development that were involved in OsEIN2-dependent ERGs as revealed by gene ontology (GO) enrichment analysis. Among these 49 genes, the function of most genes have not been reported; only *OsCTR2*, *OsRR1* and *OsWOX11* were reported to regulate crown root development (Zhao et al., 2009; Kitomi et al., 2011; Wang et al., 2013). OsCTR2 is a negative regulator of ethylene signaling and acts upstream of OsEIN2 (Wang et al., 2013); OsRR1 is a negative regulator of cytokinin signaling and its expression is repressed in *OsWOX11*-overexpressing plants (Zhao et al., 2009; Kitomi et al., 2011); OsWOX11 has been reported to control crown root development by directly regulating cytokinin signaling and homeostasis (Zhao et al., 2009; Zhao et al., 2015; Geng et al., 2022). Based on these studies, we chose OsWOX11 for further investigations. We have included these information in the revised manuscript.

3. The authors proposed that the transcriptional activation of OsWOX11 by ethylene-OsEIL1 mediates the ethylene-induced crown root development. However, in Supplemental Figure 5, the crown root development of OsWOX11 overexpression lines, which the transcription levels are not regulated by ethylene, is still induced by ethylene. These results indicate other regulations or other downstream genes rather than OsWOX11 transcriptional regulation in mediating ethylene-induced crown root development.

Response: Thanks for your important suggestions. Ethylene treatment stimulates *OsWOX11* transcription and crown root development, and overexpressing *OsWOX11* resulted in an increase in crown root number indicating that *OsWOX11* transcriptional regulation is involved in ethylene-stimulated crown root development. The crown root number of the *oswox11* mutants remained unchanged by ethylene treatment as compared to the untreated control (Supplemental Figures 8C and 8D), revealing that a

functional OsWOX11 is required for ethylene-stimulated crown root development. Ethylene has a similar effect on the number of crown roots in wild type and *OsWOX11* overexpression lines suggesting that other regulations or other downstream genes were also involved in ethylene-stimulated crown root development. All these results indicate that ethylene effects on crown roots require a functional OsWOX11, and the effect of ethylene on crown root development occurs through multiple pathways, including the OsWOX11-mediated pathway. We have added these in the revised manuscript.

4. Soil impedance-induced ethylene biosynthesis was firstly reported in the paper (PNAS, 2014, 111:3913-20; doi: 10.1073/pnas.1402491111). The references for Putative EIN3-binding sites (ATGTA/TACAT) are also not indicated.

Response: We have cited the paper (PNAS, 2014, 111:3913-20; doi: 10.1073/pnas.1402491111) and added the references for Putative EIN3-binding sites (ATGTA/TACAT) (Yang et al., 2015) in the revised manuscript.

5. The authors proposed that OsEIL1 activates OsWOX11 gene expression in mediating compacted soil-induced crown root development. The gene expression levels of OsWOX11 in WT and ethylene signaling mutants under compacted soil conditions should be examined.

Response: Thank you for your constructive suggestion. We examined the gene expression levels of *OsWOX11* in WT and ethylene signaling mutants under compacted soil conditions. Our results showed that soil compaction increased *OsWOX11* transcript levels in Nip plants. However, this effect was weakened in *ein2* and *eil1* plants, indicating that OsEIL1 activates *OsWOX11* gene expression in mediating compacted soil-induced crown root development. We have added these results in the revised manuscript.

Reviewer #3 (Comments for the Author):

In this manuscript, the authors propose a mechanistic model where ethylene and soil compaction-triggered ethylene promote crown root formation in rice through the activation of OsWOX11. Although the data presented are mostly consistent with the proposed mechanism, in my opinion, the causal link between the ethylene-induced OsWOX11 and the promotion of crown root formation has not formally been proven. As I elaborate below, some additional experiments should be performed to test the authors' hypothesis further.

Response: We greatly appreciate your constructive comments and guidance throughout the reviewing process. Based on your suggestion, some additional experiments were performed (as elaborate below). In the present manuscript, we propose that the effect of ethylene on crown root development might occur through multiple pathways, including the OsWOX11-mediated pathway where ethylene primarily regulates the transcriptional activation of OsWOX11. We have added these results and modified our statement and improved the model in the revised manuscript.

From the results displayed in Figure 2, it seems that in the ein2 mutant there are many genes regulated by ethylene that were not affected by this hormone in the wild-type plant. This needs to be explained. Similarly, I do not understand the diagram in Figure 2C diagram. The EIN2-dependent ethylene-regulated genes should be a subset of all the ethylene-regulated genes. However, the set of is much bigger than that of the ethylene-regulated genes.

Response: Thanks for your suggestions. Due to the low transcript abundance of many genes in the *ein2* mutant, even a minor fluctuation can lead to a significantly differential expression. This might results in that genes regulated by ethylene in *ein2* mutant were not affected by ethylene in the wild-type plant. We have added this explanation in the revised manuscript.

EIN2-dependent ERGs are ERGs that no longer respond to ethylene or exhibit an opposite ethylene response pattern in *ein2* mutant compared with Nip. Therefore, the EIN2-dependent ERGs should be a subset of all the ethylene-regulated genes. In Figure 2C diagram, we mistakenly refer to genes that were not affected by ethylene in

the *ein2* mutant as OsEIN2-dependent ERGs. We have corrected this mistake in the revised manuscript.

I don't think the GO analysis in Figure 2D is very helpful regarding the main topic of the paper. Perhaps this should be moved to supplemental information.

Response: Thanks for your useful suggestion, we have moved Figure 2D to supplemental information.

The functional category of genes associated with lateral root formation is not listed. Was this functional category not enriched?

Response: We only listed the top 10 functional category of cellular component, biological process, and molecular function, genes associated with lateral root formation is enriched but not involved in top 10 functional category, thus it is not listed.

All 49 genes selected and displayed in Figure 2E are regulated by ethylene and involved in root development. Why was OsWOX11 chosen?

Response: Gene ontology (GO) enrichment analysis showed that 49 genes associated with root development were involved in OsEIN2-dependent ERGs. Among these 49 genes, the function of most genes has not been reported; only *OsCTR2*, *OsRR1* and *OsWOX11* were reported to regulate crown root development (Zhao et al., 2009; Kitomi et al., 2011; Wang et al., 2013). OsCTR2 is a negative regulator of ethylene signaling and acts upstream of OsEIN2 (Wang et al., 2013); OsRR1 is a negative regulator of cytokinin signaling and its expression is repressed in *OsWOX11*-overexpressing plants (Zhao et al., 2009; Kitomi et al., 2011); OsWOX11 has been reported to control crown root development by directly regulating cytokinin signaling and homeostasis (Zhao et al., 2009; Zhao et al., 2015; Geng et al., 2022). Based on these studies, we chosen OsWOX11 for further investigations. We have added these in the revised manuscript.

The oswox11-c2 mutation results in a frameshift and, I assume, a premature stop codon. This is not mentioned in the text or shown in Supplemental Figure 4B.

Response: The *oswox11-c2* mutant contained 1-bp insertion in the coding region, triggering a frame shift in the open reading frame, but did not result in a premature stop codon. We have included this in the updated figure in the revised manuscript.

There is no correlation between the OsWOX11 expression levels and the number of crown roots. This needs to be discussed. Similarly, although the c1 and c3 mutant alleles are practically identical (just one aa different), the number of crown roots is significantly different. This also needs to be explained.

Response: We examined the OsWOX11 protein in *OsWOX11* overexpression lines and found that although the expression of *OsWOX11* was significantly increased in *OsWOX11-OX* plants, whereas the OsWOX11 protein was increased slightly and showed no changes in *OsWOX11-OX* lines, implying that OsWOX11 is a key regulator in plant development and plants must maintain proper levels of OsWOX11 protein to ensure normal growth. This is why there is no correlation between the *OsWOX11* expression levels and the number of crown roots. In the present study, crown root number of 10-d-old plants was counted. We further observed the number of crown roots of *c1* and *c3* mutant at the three-leaf stage and heading stage, and the results showed that *c1* and *c3* mutant had similar crown root number. Thus, the different crown root number of 10-d-old *c1* and *c3* mutant might be due to the difference of seed vigor. We have added these results in the revised manuscript.

The fact that ethylene regulates OsWOX11 in an EIN2- and EIL1-dependent manner does not mean that the ethylene effects on the number of crown roots are mediated by an increase in the levels of OsWOX11. In fact, the observation that ethylene has a similar effect on the number of crown roots in wild type and OsWOX11 overexpression argues against the idea that the ethylene effect on crown roots is due to an increase in the levels of OsWOX11. In the OsWOX11 overexpression lines, the transcript levels are 12-fold higher (Figure S4C) than in the wild type but only slightly

increase the number of crown roots (Figure S5B). On the other hand, ethylene treatment has a similar or lower effect on the transcript levels of OsWOX11 (Figure S7A) but a much greater effect on the number of crown roots (Figure S5B). I think the results indicate that the ethylene effects on crown roots require a functional OsWOX11 but do not support the model where this ethylene effect is simply mediated by the transcriptional activation of OsWOX11 by ethylene.

Response: Thanks for your important suggestions, we agree with your hypotheses that the ethylene effect is not simply mediated by the transcriptional activation of OsWOX11.

Based on your suggestion and our findings (listed below) in the present manuscript, we propose that the effect of ethylene on crown root development might be occurred through multiple pathways, including the OsWOX11-mediated pathway that ethylene is primarily mediated by the transcriptional activation of OsWOX11. We have modified our statement and the model in the revised manuscript.

Firstly, ethylene treatment stimulates OsWOX11 transcription and crown root development, and OsEIL1 directly binds to the *OsWOX11* promoter to activate its expression.

Secondly, overexpressing *OsWOX11* resulted in an increase in crown root number and genetic analysis indicates that ethylene signaling acts upstream of the OsWOX11-mediated pathway to regulate crown root development, indicating that the transcriptional activation of *OsWOX11* is required for ethylene-induced crown root development.

Furthermore, the crown root number of the *oswox11* mutants remained unchanged under ethylene treatment as compared to the untreated control, revealing that a functional OsWOX11 is required for ethylene-stimulated stimulates crown root development.

Related to the previous point, to test the model where ethylene-mediated upregulation of OsWOX11 is responsible for the observed increase in the expression of OsCKX4 and the reduction in the levels of OsRR2, the authors should check whether or not the

ethylene effects on the expression of these two genes are blocked in the oswox11 mutants.

Response: Thank you for your suggestion. We checked the expression of *OsCKX4* and *OsRR2* in the *oswox11* mutant, our results showed that ethylene treatment suppressed *OsRR2* expression and induced *OsCKX4* expression in Nip plants, and this effect was weakened in *oswox11* mutant, indicating that ethylene-mediated upregulation of *OsWOX11* is responsible for the increase in the expression of *OsCKX4* and the reduction in the levels of *OsRR2*. We have added these results in the revised manuscript.

As indicated before, the fact that ethylene has a significant effect in the OsWOX11 overexpression lines suggest that the ethylene effect is not simply due to an increase in the transcription of OsWOX11. To shed further light on the validity of the presented model, the authors should test the effects of the oseil1 and/or osein2 mutations on the number of crown roots in the OsWOX11 overexpression lines. The expectation, according to the authors' model, is that the mutations in oseil1 and osein2 should have no effect on the number of crown roots present in the OsWOX11 overexpression lines. On the other hand, an effect of the oseil1 or osein2 mutants would suggest a more complex scenario where ethylene may affect the number of crown roots by a mechanism other than the transcriptional activation of OsWOX11. In this same line of thought, the analysis of the ethylene effects on the transcript levels of OsRR2 and OsCKX4 in the oswox11 mutant should also help to better define the mechanism by which ethylene regulates crown root number.

Response: Thanks for your constructive suggestions. In the present study, we showed that ethylene transcriptionally activates the expression of *OsWOX11* primarily mediating the ethylene signaling pathway and OsEIL1 directly binds to the *OsWOX11* promoter to activate its expression. Overexpressing *OsWOX11* resulted in an increase in crown root number and genetic analysis indicates that ethylene signaling acts upstream of the OsWOX11-mediated pathway to regulate crown root development. These results indicate that the transcriptional activation of *OsWOX11* is required for

ethylene-induced crown root development. The crown root number of the *oswox11* mutants remained unchanged under ethylene treatment as compared to the untreated control (Supplemental Figures 8C and 8D), indicating that ethylene effects on crown roots require a functional OsWOX11. We also checked the expression of *OsCKX4* and *OsRR2* in the *oswox11* mutant with or without ethylene treatment. Our results showed that ethylene treatment suppressed *OsRR2* expression and induced *OsCKX4* expression in Nip plants, and this effect was weakened in *oswox11* mutant, indicating that ethylene-mediated upregulation of *OsWOX11* is responsible for the increase in the expression of *OsCKX4* and the reduction in the levels of *OsRR2*, and *OsWOX11*-mediated pathway is involved in ethylene-regulated crown root development. All these results indicate that the effect of ethylene on crown root development might be occurred through multiple pathways, including the OsWOX11-mediated pathway that ethylene is primarily mediated by the transcriptional activation of OsWOX11.We have added these results and modified the model in the revised manuscript. We also generated *eil1 OsWOX11-OX* and *ein2 OsWOX11-OX* plants by crossing *OsWOX11-OX-1* with *eil1* or *ein2*, yet the generation of plant materials is time-consuming, we have not obtained the *eil1 OsWOX11-OX* and *ein2 OsWOX11-OX* plants yet.

Are the effects on grain size triggered by the overexpression of OsEIL1 suppressed in the oswox11 mutant? Grain size should be properly quantified and proper statistical methods applied.

Response: Thanks for your suggestions. We observed the grain size of Nip, *oswox11-c1*, *EIL1-OX*, and *EIL1-OX oswox11-c1* plants, and the results showed that the effects on grain size triggered by the overexpression of OsEIL1 suppressed in the *oswox11* mutant. We have added these results in the revised manuscript. Moreover, grain size was quantified and statistical methods were applied in the revised manuscript.

In this work, the authors only show grain size, but not yield. Including yield results

will be relevant to evaluating the potential biotechnological applications of these findings claimed by the authors.

Response: Thanks for your suggestions. We have added the thousand-grain weight and grain yield per plant in the revised manuscript.

Other minor points:

The constructs used in the dual luciferase assay should be indicated in the materials and methods section.

Response: The 2.0 kb *OsWOX11* promoter region was cloned into pGREENII0800 vector (*OsWOX11p:LUC*), and the effector was generated by inserting the coding sequence of OsEIL1 into pCAMBIA1307 plasmid under control of the *35S* promoter (*35S:OsEIL1*). We have added this in the materials and methods section in the revised manuscript.

To further confirm that, indeed, the oswox11 mutations suppress the effects of OsEIL1 overexpression, the levels of expression of OsEIL1 in the double mutant should be examined to confirm they are similar to those of the original overexpression line as the presence of a transgene in the oswox11 may be causing silencing of the OsEIL1 transgene.

Response: The expression of *OsEIL1* in the *EIL1-OX oswox11-c1* plants was examined and the results showed that they are similar to those of the original overexpression line. We have added these results in the revised manuscript.

Dear Dr. Hua Qin:

We have received reviews of your manuscript entitled "OsEIL1-OsWOX11 module is required for crown root development in response to soil compaction." On the basis of the advice received, the board of reviewing editors would like to accept your manuscript for publication in The Plant Cell. This acceptance is contingent on revision based on the comments of our reviewers. In particular, please consider the the suggestions by Reviewer #3.

Notes from the Editor-in-Chief:
We are trying to make a concerted effort to change green/red comparisons to green/magenta to make our figures understandable to those with color vision deficiencies. We noticed that a small number of your figures utilize red/green contrasts, so as you prepare the final version of the figures, please check the figures for red/green color use. Magenta, blue, yellow, orange are all good alternatives. It was the heat maps in Figure 2 that need this change; you might want to check over the Supplemental Figures for similar comparisons. Please note that color changes do not need to be highlighted or tracked in the revised manuscript, but could be noted in the cover letter or response document.

Also, you have used the default colors from an older version of Excel for many of your bar and line or pie graphs. We generally discourage this, also because it includes red and green that are not easily distinguished, but also there are more 'modern' color palettes available that look less dated.
Please see this PDF document that includes guidelines for figures - the examples included here may be a useful template for how your charts or figures could be reformatted, including color schemes that are modern and easy to understand:
https://tpc.msubmit.net/html/TPC_Detailed_Figure_Guidelines.pdf

Please highlight all changes and include a detailed annotation of changes of the text, with line numbers, and noting your responses to the comments.

To submit your revised manuscript, click:

Link Not Available

If you have any questions about the revision submission procedures, please contact the Editorial Office Staff (tpc-submissions@aspb.org). If you cannot return the revised manuscript within 30 days, please let us know. Otherwise, we will assume that you have elected not to revise the manuscript and withdraw it.

Thank you very much for the privilege of reviewing this work. I look forward to receiving the next version.

Sincerely,

The Plant Cell Board of Editors

Reviewer #1 (Comments for the Author):

I am happy with the revisions, and have no further comments.

Reviewer #2 (Comments for the Author):

The revised manuscript is improved. Most of my concerns have been properly addressed. I don't have further questions about it.

Reviewer #3 (Comments for the Author):

The authors have satisfactorily addressed most of my previous comments and concerns. There is one point that I think the authors should further consider.
The authors explain that the cause for the reported ethylene effect on the transcript levels in many genes and that many of these changes do not happen in the corresponding wildtype is that the corresponding genes are very low expressed in ein2. I

suggest that the authors include an expression level threshold filter in their gene expression analysis. Otherwise, the readers may be misled to believe that ethylene has a strong transcriptional response in the ein2 mutant.

Please also note the following:

-The Plant Cell now requires authors to complete and submit an author revisions checklist upon submission of a revised manuscript. The aim of the checklist is to aid authors in preparing a high-quality manuscript, facilitate the review and assessment of revised manuscripts, and help to ensure that journal standards are maintained across the board. If your manuscript is accepted, the completed checklist will be published as supplemental material attached to the article online. Please download a copy of the checklist (pdf fillable form) at this link, for submission with your revised manuscript: https://tpc.msubmit.net/html/Author_Revisions_Checklist.pdf.

-Supplemental materials should be restricted to large datasets and tables, presentation of replicates, and validation of reagents, methods, or genotypes. Any data that are used to support the major claims must be in the main manuscript. Supplemental figure legends must indicate what figure in the main manuscript is supported by the supplemental data presented. Please justify how each of the supplemental figures meet the criteria.

-Sampling methods and the nature of "biological replicates" should be described precisely (i.e. different plants, parts of plants, pooled tissue, independent pools of tissue, sampled at different times, etc.), along with a clear description of and rationale for any statistical analyses conducted. The reader should know exactly what was sampled; what forms the basis of the calculation of any means and statistical parameters reported. This is also necessary to ensure that proper statistical analysis was conducted.

Reviewer #1 (Comments for the Author):

I am happy with the revisions, and have no further comments.

Response: Thanks very much for your positive comments.

Reviewer #2 (Comments for the Author):

The revised manuscript is improved. Most of my concerns have been properly addressed. I don't have further questions about it.

Response: Thanks very much for your positive comments.

Reviewer #3 (Comments for the Author):

The authors have satisfactorily addressed most of my previous comments and concerns. There is one point that I think the authors should further consider.

The authors explain that the cause for the reported ethylene effect on the transcript levels in many genes and that many of these changes do not happen in the corresponding wildtype is that the corresponding genes are very low expressed in ein2. I suggest that the authors include an expression level threshold filter in their gene expression analysis. Otherwise, the readers may be misled to believe that ethylene has a strong transcriptional response in the ein2 mutant.

Response: Thank you very much for your constructive suggestion. We filtered out genes with expression level less than 0.1 in our gene expression analysis and modified Figures 2A and 2B accordingly in the revised manuscript, which could avoid misled readers to believe that ethylene has a strong transcriptional response in the *ein2* mutant.

Dear Dr. Hua Qin:

We are pleased to inform you that your paper entitled "OsEIL1-OsWOX11 module is required for crown root development in response to soil compaction" has been accepted for publication in The Plant Cell, pending a final editorial review by a science editor. At this stage, your manuscript will be evaluated by a science editor with respect to its presentation of scientific content, compliance with journal policies, and presentation for a broad readership. The Plant Cell has contracted with Plant Editors (planteditors.com) to provide this service to our authors, and you will soon receive additional information on this process.

Please note that each author needs to link their ORCID identifier to their account in the system before your manuscript can be published. If any authors do not have an ORCID linked to their account, they will receive a message with a link to complete this task. Please ensure that ALL of your coauthors have completed this task as soon as possible.

ASPB offers an OPEN option that allows authors to have their online articles available for free to all users immediately upon publication. For more information about the ASPB OPEN option, refer to the Final Submission Checklist Form.

The Plant Cell and The Arabidopsis Information Resource (TAIR) are collaborating to collect functional annotation data about Arabidopsis genes from authors. This includes information about the gene's molecular function (e.g., kinase activity, ATP synthetase activity), the biological process/es it is involved in (e.g., endosperm development, threonine biosynthesis), its subcellular location (e.g., nucleus, ER), anatomical or developmental expression pattern (e.g., leaf, ovule, flower stage 10, seedling stage), or its partner in a protein-protein interaction (e.g., AT1G01010 interacts with AT1G01020).

If your paper contains results falling into one or more of these categories for Arabidopsis genes, we request that you now submit these data for inclusion in TAIR by filling in the form provided at the following URL: https://www.arabidopsis.org/doc/submit/functional_annotation/123. If you need further clarification on what types of data can be submitted please contact curator@arabidopsis.org.

Finally, we encourage your submission of artwork for the journal cover. Monthly issues will have an online cover image and selected covers will be used for posters, other promotional items, and "wallpaper" for mobile devices. For more information, refer to Cover Submission in the Instructions for Authors https://tpc.msubmit.net/cgi-bin/main.plex?form_type=display_auth_instructions.

We look forward to seeing your paper published.

Sincerely,

The Plant Cell Board of Editors

=====
IMPORTANT REMINDER: PEER REVIEW REPORTS
=====

If you opted to publish a peer review report along with your article during the original submission process, it will be prepared by the editorial staff and publicly posted with your manuscript, inside the zip file that contains any other supplemental material. As a reminder, the peer review report is a public record of all comments from editors and reviewers, as well as your prior responses, as you received them in the decision letters for each draft of your manuscript. If you agreed to publish this report and have changed your mind, or are not sure if you selected this option, please contact the editorial office as soon as possible before signing the license agreement from our publisher.

=====

----FOR ASPB OFFICE USE ONLY (DO NOT EDIT)----
MSID: 37787
Corresponding Author: Dr. Qin
